# School-age growth and development following infant feeding and/or water, sanitation, and hygiene interventions in rural Zimbabwe: long-term follow-up of a cluster-randomised trial

**DOI:** 10.1016/j.eclinm.2024.102946

**Published:** 2024-11-22

**Authors:** Joe D. Piper, Clever Mazhanga, Marian Mwapaura, Gloria Mapako, Idah Mapurisa, Tsitsi Mashedze, Eunice Munyama, Maria Kuona, Thombizodwa Mashiri, Kundai Sibanda, Dzidzai Matemavi, Monica Tichagwa, Soneni Nyoni, Asinje Saidi, Manasa Mangwende, Gabriel Mbewe, Dzivaidzo Chidhanguro, Eddington Mpofu, Joice Tome, Batsirai Mutasa, Bernard Chasekwa, Handrea Njovo, Chandiwana Nyachowe, Mary Muchekeza, Virginia Sauramba, Melissa J. Gladstone, Jonathan C. Wells, Elizabeth Allen, Lawrence H. Moulton, Melanie Smuk, Jean H. Humphrey, Lisa F. Langhaug, Naume V. Tavengwa, Robert Ntozini, Andrew J. Prendergast

**Affiliations:** a Zvitambo Institute for Maternal and Child Health Research, Harare, Zimbabwe; b Blizard Institute, Queen Mary University of London, London, UK; c London School of Hygiene and Tropical Medicine, London, UK; d Ministry of Health and Child Care, Harare, Zimbabwe; e Institute of Translational Medicine, University of Liverpool, Liverpool, UK; f Population Policy and Practice Research and Teaching Department, UCL Great Ormond Street Institute of Child Health, London, UK; g Department of International Health, Johns Hopkins Bloomberg School of Public Health, Baltimore, MD, USA

**Keywords:** Stunting, Child, Development, WASH, Nutrition

## Abstract

**Background:**

Few trials have explored long-term effects of interventions designed to reduce child stunting. We evaluated school-age outcomes in rural Zimbabwean children who received cluster-randomised water, sanitation and hygiene (WASH) and/or infant and young child feeding (IYCF) interventions from pregnancy up to 18 months of age.

**Methods:**

The Sanitation Hygiene Infant Nutrition Efficacy (SHINE) trial enrolled pregnant women from two rural Zimbabwean districts (Chirumanzu and Shurugwi) between 2012 and 2015, and cluster-randomised them using a 2 × 2 factorial design to standard-of-care, WASH, IYCF, or combined WASH & IYCF, with a co-primary outcome of height-for-age Z-score and haemoglobin at 18 months (clinicaltrials.govNCT01824940). Children who were HIV-unexposed, age 7 years, and still living in Shurugwi district were eligible for this follow-up study (registered at PACTR 202201828512110) and measured between 1st April 2021 and 30th September 2022. The primary outcome at 7 years was cognitive function using the Kaufman Assessment Battery for Children (KABC-II). Secondary outcomes were executive function, literacy and numeracy, fine motor skills, socioemotional function, handgrip strength, broad jump distance, shuttle-run test distance, anthropometry, lean mass index, and skinfold thicknesses. Study nurses conducting assessments were blinded to intervention arm. Analysis followed a pre-registered statistical analysis plan. Intention-to-treat analyses using generalized estimating equations were used to assess the long-term effects of WASH and IYCF on each outcome, leveraging the factorial trial design. A pre-specified subgroup analysis by child sex was also performed.

**Findings:**

Among 3989 HIV-negative women, 3676 children were assessed at age 18 months; of these, 1002 (510 female) were assessed at mean (SD) age 7.3 (0.2) years. There was no effect of IYCF or WASH on the KABC-II score or secondary cognitive outcomes, except a small improvement in socioemotional function in WASH arms (−0.98 points, 95% CI −1.73, −0.22, p = 0.01). Children in IYCF arms had higher handgrip strength (0.28 Kg, 95% CI 0.02, 0.53, p = 0.03); however, in the pre-specified subgroup analysis, improved handgrip strength was seen only in boys (0.53 Kg, 95% CI 0.19, 0.87 p = 0.002). There were no significant effects of either intervention on other outcomes.

**Interpretation:**

Early-life IYCF and WASH led to few functional benefits by school-age. Interventions that are more comprehensive, delivered for longer, and include nurturing care should be considered to improve long-term cognitive and physical function.

**Funding:**

10.13039/100004440Wellcome [220671/Z/20/Z, 108065/Z/15/Z]; 10.13039/100000002NIH [R61HD103101]; Thrasher [15250]; and 10.13039/100014248IMMANA [3.02].

**Research in context**

**Evidence before this study**

Approximately 150 million children are at risk of poor linear growth or stunting and 250 million remain at risk of impaired cognitive development. Pubmed, Medline, Embase, Global Health and Google Scholar were searched including key search concepts relating to stunting, interventions, nutrition, water, sanitation and hygiene (WASH) AND (Child) AND growth or development or strength or function performed from the 5th October 2019 and updated until 3rd March 2021, and then updated later in December 2021. A meta-analysis examining the effects of interventions on cognition and growth showed that nutritional supplements had small benefits for linear growth under 6 years and cognition. For WASH, the evidence remains less clear, although individual studies have shown occasional impact.

Small-quantity lipid-based nutrient supplements (SQ-LNS) are the most effective nutrition interventions and are designed to complement the diets of children aged 6–24 months, using a formulation suitable for programmatic scale-up. Recent meta-analyses have shown that SQ-LNS increases child growth and haemoglobin at 6–24 months of age, with a smaller effect on early child development. The Sanitation Hygiene Infant Nutrition Efficacy (SHINE) cluster-randomised trial contributed to these meta-analyses and showed a similar benefit of SQ-LNS at 18-months for growth and haemoglobin, but no effects on neurodevelopment.

Mid-childhood reflects both early-life environmental conditions and predicts later adult function. However, this period of childhood has been termed the ‘missing middle’ due to the lack of data on long-term follow-up from early-life interventions. One exception is the INCAP trial in 1970's Guatemala, which showed that the small effects of protein supplementation on growth between 0 and 24 months was associated with subsequent benefits in school achievement and adult earnings. One early-life handwashing study in Karachi, Pakistan, showed a benefit on cognition at school-age, and one study that reduced open defecation in India was associated with an increase in later school-age cognition. There is also some evidence of benefit in growth and cognition from the WASH Benefits study in Bangladesh but not in Kenya. However, long-term follow-up studies are limited and very few have investigated multiple outcomes to examine the holistic effect on child growth and function.

**Added value of this study**

This is the first long-term follow-up of a cluster-randomised trial of early-life nutrition and WASH in sub-Saharan Africa, which holistically measured growth, cognitive and physical function using contextually adapted tools. Children were assessed more than 5 years after the trial interventions had ended. Overall, there were few benefits of early-life nutrition or WASH interventions by school age in this first study to measure long-term follow-up.

**Implications of all the available evidence**

We found minimal effects of early-life IYCF or WASH interventions on cognitive function, growth, body composition, or physical strength by school-age. This study highlights that the modest early-life growth benefits from IYCF interventions may not translate into long-term gains in cognitive or physical function. Collectively, these findings suggest that additional interventions, which are started earlier, provided for longer, and more comprehensively enhance the child's environment, may be required to sustainably improve long-term cognition and physical function in sub-Saharan Africa.

## Introduction

Stunting affects 22% (149 million) children under 5 years^1^ and is associated with increased child mortality, long-term cognitive and health deficits, poorer school performance, and lower adult earnings.^2^ Stunting is defined as a height-for-age Z-score (HAZ) more than two standard deviations below the global reference standard,^3^ although linear growth faltering affects many more children above this cutoff.^3^ Improved infant and young child feeding (IYCF)^4^ during the period from 6 to 24 months of age is the most effective current intervention, but only increases linear growth modestly (+0.11 HAZ). Daily small-quantity lipid-based nutrient supplements (SQ-LNS) during the period of complementary feeding reduce stunting by 12%.^5^^,^^6^ There is continued interest in the hypothesis that improved water, sanitation and hygiene (WASH) may improve growth.^7^ Despite this, most trials have not found improvements in growth following household- or community-level WASH interventions.^8^

Globally, 43% of children (250 million) are at risk of not reaching their full developmental potential due to stunting and severe poverty. A recent systematic review showed early-life IYCF interventions can improve neurodevelopment,^9^ and SQ-LNS increases child development scores by approximately 1–1.5 IQ points, particularly in settings with a high stunting burden. However, overall effects of nutrition interventions on neurodevelopment are small, and growth is not a reliable proxy for functional outcomes such as cognition.^10^ Water, sanitation and hygiene (WASH) interventions that reduced diarrhoea showed impacts on early child development in the WASH Benefits trial in Bangladesh,^11^ and on school-age cognition following a handwashing intervention in Pakistan,^12^ but other studies have shown no consistent effects.^13^, ^14^, ^15^

There has been a recent call for long-term follow-up of IYCF and WASH intervention trials^16^ that combine both growth and neurodevelopment assessments. School-age follow-up is crucial in determining if early-life intervention effects are lost, sustained, or provide greater than expected long-term benefits by altering the trajectory of child growth and development. Assessment at school-age enables the measurement of more complex outcomes of neurodevelopment, including cognitive processing, executive function, school performance, and socio-emotional behaviour. In addition, body composition, physical strength and fitness provide a holistic measurement of child function at school age,^17^ which is predictive of later adult outcomes. The Sanitation Hygiene Infant Nutrition Efficacy (SHINE) trial^18^ recruited pregnant mothers and randomised their infants to improved IYCF and/or improved WASH in rural Zimbabwe. The trial showed a small benefit of IYCF on growth and haemoglobin at age 18 months,^13^ but no early-life benefit of WASH on growth^13^; neither intervention impacted neurodevelopment at age 2 years.^14^ However, it is possible the growth benefits from IYCF could have been amplified by school-age with additional benefits in function. Therefore, here we report 7-year growth, physical function, and cognitive outcomes for children who received standard of care (SOC), improved IYCF and/or WASH interventions in early life within the SHINE trial.^18^

## Methods

### Overview of the original SHINE trial

The Sanitation Hygiene Infant Nutrition Efficacy (SHINE) trial design is described elsewhere.^18^ Briefly, SHINE was a 2 × 2 factorial cluster-randomised trial of improved IYCF and/or improved WASH with a co-primary outcome of length-for-age Z-score and haemoglobin at age 18 months. The trial was conducted in two districts of rural Zimbabwe (Chirumanzu and Shurugwi) divided into 212 clusters, defined as the catchment area of 1–4 community health workers (CHW) employed by the Ministry of Health and Child Care (MoHCC).^19^ Clusters were randomly assigned to one of four treatment arms using highly constrained randomisation: standard-of-care (SOC), IYCF, WASH and combined IYCF + WASH. Between November 2012 and March 2015, women were enrolled if they were confirmed pregnant, were a permanent resident in the study cluster, and provided written informed consent.

Interventions were developed through extensive formative work and piloting and delivered by CHWs during home visits,^18^^,^^20^, ^21^, ^22^ with supportive supervision to assess implementation fidelity. The content of CHW visits varied by arm^19^:

1) **SOC:** CHWs encouraged early, exclusive and prolonged breastfeeding,^21^ promoted family planning, immunisation and prevention of mother-to-child-transmission (PMTCT) of HIV;
2) **WASH:** In addition to SOC interventions, modules promoted safe disposal of faeces, handwashing with soap, protection of infants from geophagia, drinking chlorinated water, and hygienic preparation of complementary food. A Blair ventilated improved pit latrine was constructed, two ‘tippy-tap’ handwashing stations were installed, and monthly deliveries of liquid soap and water chlorination solution (WaterGuard, Nelspot, Zimbabwe) provided until 18 months postpartum. A plastic baby mat and playpen were provided to protect from geophagia.
3) **IYCF:** CHWs delivered the SOC interventions plus additional modules promoting nutritious infant diets using local foods and frequent responsive feeding during illness. From 6 to 18 months postpartum, a daily 20g sachet of SQ-LNS (Nutriset, Malaumay, France) was provided to add to complementary food.
4) **WASH + IYCF:** CHWs delivered all SOC, WASH and IYCF interventions.

Masking of interventions was not possible due to their nature. A latrine was constructed for each household in the non-WASH arms (i.e. IYCF and SOC arms) after the 18-month visit.

At baseline, mothers had weight, height, mid-upper arm circumference (MUAC) and haemoglobin measured and were tested for HIV using a rapid test algorithm, then offered further testing at 32 gestational weeks and 18 months postpartum. Women with HIV were excluded from the current analysis since the trial design pre-specified that all outcomes would be stratified by maternal HIV status. A baseline questionnaire measured food insecurity (Coping Strategies Index), household minimum dietary diversity, household wealth,^23^ and maternal capabilities including gender norms, depression, and social support.^24^ Birth details including birth date, weight and delivery details were obtained from health records. Infant weight, length, head circumference and MUAC were measured at postnatal visits between 1 and 18 months of age. Intervention compliance was assessed at all visits through maternal report and structured observations, as previously described.^19^

As previously reported,^13^ IYCF improved length-for-age Z-score by 0.16 (95% CI 0.08, 0.23) at age 18 months among children born to HIV-negative mothers, and reduced stunting by 21%, while WASH had no evidence of an effect on growth.^13^ IYCF also increased child haemoglobin by 2.03 g/L (95% CI 1.28, 2.79), but WASH had no effect.^13^ There was no significant impact of the SHINE IYCF or WASH interventions on child neurodevelopment at 2 years of age.^14^

### Study design and participants

To evaluate the long-term effects of IYCF and WASH on child health outcomes, we conducted a substudy to assess child growth, body composition, physical and cognitive function at 7 years of age in one of the two original study districts (Shurugwi). No further interventions had been provided after age 18 months. The protocol, trial design, and statistical analysis plan for the follow-up study are registered at https://osf.io/8e2zh. In brief, children were eligible if they were aged 7–8 years, still resident in Shurugwi district, and born to HIV-negative mothers who were willing to provide written informed consent. Among children born to HIV-negative mothers and evaluated at the trial endline at age 18 months in Shurugwi district, 250 per intervention arm meeting the eligibility criteria were randomly selected by computer using the *sample* program in Stata 13. Children who were no longer resident in Shurugwi, with an unknown maternal pregnancy HIV status, or outside the age window, were ineligible. Children who were unable to be visited or whose families declined participation were replaced randomly by another eligible child from the same trial arm. Measurements were performed between 1st April 2021 and 30th September 2022.

### Procedures

CHWs first approached each family to ascertain if the child was available and the household was interested. Written informed consent from the primary caregiver and written assent from the child were obtained by research nurses, following a discussion and a demonstration of the tools used to conduct the measurements.

Assessments were performed by primary care nurses extensively trained and supervised in the study measurement techniques, during a single home visit using two tents pitched in or close to the homestead. We developed, piloted and then deployed the School-Age Health, Activity, Resilience, Anthropometry and Neurocognitive (SAHARAN) toolbox for all assessments, as described previously.^17^ Briefly, SAHARAN consists of a caregiver questionnaire, child questionnaire, and direct tests undertaken with the child to assess cognitive function, growth and physical function. All tests had standardised explanations, demonstrations, and translations in local languages. Data were collected electronically using Open Data Kit (opendatakit.org) on Android tablets (Samsung Galaxy Tab A), or on paper with subsequent data entry into ODK. Research nurses underwent 6-monthly refresher training and standardisation (see Appendix), and supportive supervision was provided through regular observed visits by a paediatrician (JDP).

All primary caregivers were offered repeat HIV testing, unless they had a documented negative test result in the previous 3 months. If the mother had acquired HIV since the end of the trial, declined testing, or was unavailable, HIV testing of the child was undertaken with age-appropriate assent using role plays. The Determine HIV-1/2 rapid test (Abbott) was used for initial testing; positive results were repeated using the HIV-1/2 Stat-Pak rapid test (Chembio). Children with HIV were removed from the current analysis and referred to clinics. Mothers or caregivers with HIV were referred to local clinics.

### Outcomes

The pre-specified primary outcome was cognitive function, defined as the total score from eight subtests of the Kaufman Assessment Battery for Children 2nd edition (KABC-II; Pearson UK),^25^^,^^26^ which forms the Mental Processing Index (MPI). The KABC-II was previously extensively piloted in Zimbabwe within the SAHARAN toolbox and two of the eight subtests were adapted to improve cross-cultural validity, as published elsewhere.^27^ Secondary cognitive outcomes were assessed using the custom-developed School Achievement Test (SAT) which measures literacy and numeracy; a finger tapping test^28^ which measures fine motor skills; executive function, using three subtests from the tablet-based Plus-EF test^29^; and the caregiver-reported Strengths and Difficulties Questionnaire (SDQ), which provides a measure of the child's socioemotional function and behaviour.^30^ All these cognitive tests were previously piloted, with plausible associations observed using the SAHARAN toolbox^17^; further details are in supporting information. Supportive supervision was undertaken through weekly visits by a research nurse (CM) and 3–6 monthly visits by the study clinician (JP); standardisation exercises were performed 6–9 monthly for KABC-II, SAT, finger tapping and anthropometry (see supporting information).

Growth outcomes were height, knee-heel length, head circumference, mid-upper arm circumference (MUAC); waist, hip and calf circumferences; and weight (Seca 874 Dr scale, Germany). Body composition was assessed using Holtain calipers (Crosswell, UK) to measure central subcutaneous fat (subscapular and supra-iliac skinfold thicknesses) and peripheral subcutaneous fat (triceps and calf skinfold thicknesses), and bioimpedance analysis (BIA; Bodystat, UK) to give an impedance reading (Z). Lean mass was assessed both in absolute terms using the impedance index (height^2^/Z), and in height-adjusted terms (lean mass index, 1/Z)^31^; tissue health was assessed using the bioimpedance phase angle. Each of these outcomes avoids the need for population-specific BIA calibration equations and is robust for the comparison of groups, as used here.^32^

Physical function outcomes were handgrip strength, measured in each hand using a dynamometer (Takei, Japan); and leg strength, measured as the distance jumped in the broad jump from a standing position. Cardiovascular fitness was measured by the shuttle-run test, where the child repeatedly runs between two markers placed 20 m apart, arriving at each end before a timed beep (Beep Test, Ruval Enterprises, Canada). The child runs until they miss the beep three times in a row or withdraws from tiredness. From the maximum level achieved in the shuttle-run test, VO_2max_, which is the maximum rate at which the body uses oxygen during exercise, was calculated.^33^ Resting and post-exercise blood pressure was measured using a manual sphygmomanometer (Medisave, UK). Haemoglobin was measured (Hemocue) on a finger-prick blood sample.

The caregiver questionnaire assessed household demographics, socioeconomic status using a locally validated wealth index,^23^ maternal schooling, caregiver depression,^34^ gender norms,^35^ caregiver social support,^35^ and food insecurity^36^

### Statistics

A pre-specified Statistical Analysis Plan was registered at https://osf.io/8e2zh and the long-term follow-up protocol has been published.^37^ Analysts remained blinded to the original trial allocation until all data were collected and cleaned. Stata (versions 15 & 17) was used for all analyses.

For the primary outcome, 1000 children (500 IYCF vs 500 non-IYCF, or 500 WASH vs 500 non-WASH capitalising on the factorial design) provided 86% power to detect a 0.2 standardised effect size in the absolute MPI difference between intervention and control arms, assuming intra-cluster correlation of 0.05 and sampling from 100 clusters, with two-sided alpha of 0.05. Data collection was undertaken immediately after enrolment, therefore no allowance for loss to follow-up was required.

We compared baseline characteristics of participants enrolled in the follow-up study between trial arms while handling within-cluster correlation using multinomial regression models with robust variance estimation. All analyses were intention-to-treat at the child level, with residence at the time of consent into the original SHINE trial dictating the study arm. All the SAHARAN toolbox^17^ outcomes were continuous measures. Therefore the absolute difference in mean score between treatment groups was estimated using generalised estimating equations that accounted for within-cluster correlation, with an exchangeable working correlation structure. Although the study was not powered to detect a statistical interaction between the IYCF and WASH interventions, it was estimated for key outcomes within each domain (MPI for cognitive; HAZ for growth; and grip strength for physical function). If the interaction term was significant (p < 0.05 from a Wald test), or there was a sizeable point estimate (difference in mean score >0.25 standard deviations), we used a regression model with three dummy variables to represent the comparison of each of the three treatment arms (IYCF, WASH and IYCF + WASH) compared to the SOC arm, for all outcomes within that domain. If the interaction term was not significant, we used a regression model with two terms to represent the treatment arms: the effects of IYCF were estimated by comparing the two IYCF arms (IYCF and WASH + IYCF) with the two non-IYCF (WASH and SOC) arms, and the effects of WASH were estimated by comparing the two WASH arms (WASH and WASH + IYCF) with the two non-WASH arms (IYCF and SOC). The primary analysis was unadjusted. Adjusted analyses included prespecified covariates and additional baseline covariates based on a directed acyclic graph (see Appendix). A pre-specified subgroup analysis explored interactions with child sex; if p < 0.10 for any outcome, separate GEE models for boys and girls were used. There was minimal missing outcome data (<3%) so observations with missing outcome variables were omitted from the analysis. Although the proportion of missing covariate data in adjusted models was small (<10%), we included a categorical variable for missingness.

### Ethics

The Medical Research Council of Zimbabwe approved the study protocol (MRCZ/A/1675). The SHINE follow-up study was registered with the Pan-African Clinical Trials Registry (PACTR202201828512110). Informed consent from all participants was obtained, including consent from the primary caregiver and assent from the child.

### Role of funding source

The funder of the study had no role in study design, data collection, data analysis, data interpretation, or writing of the report. Authors JP, MM, JT, and RN had full access to the data throughout the study.

## Results

### Enrolment and follow-up

**Fig. 1:**
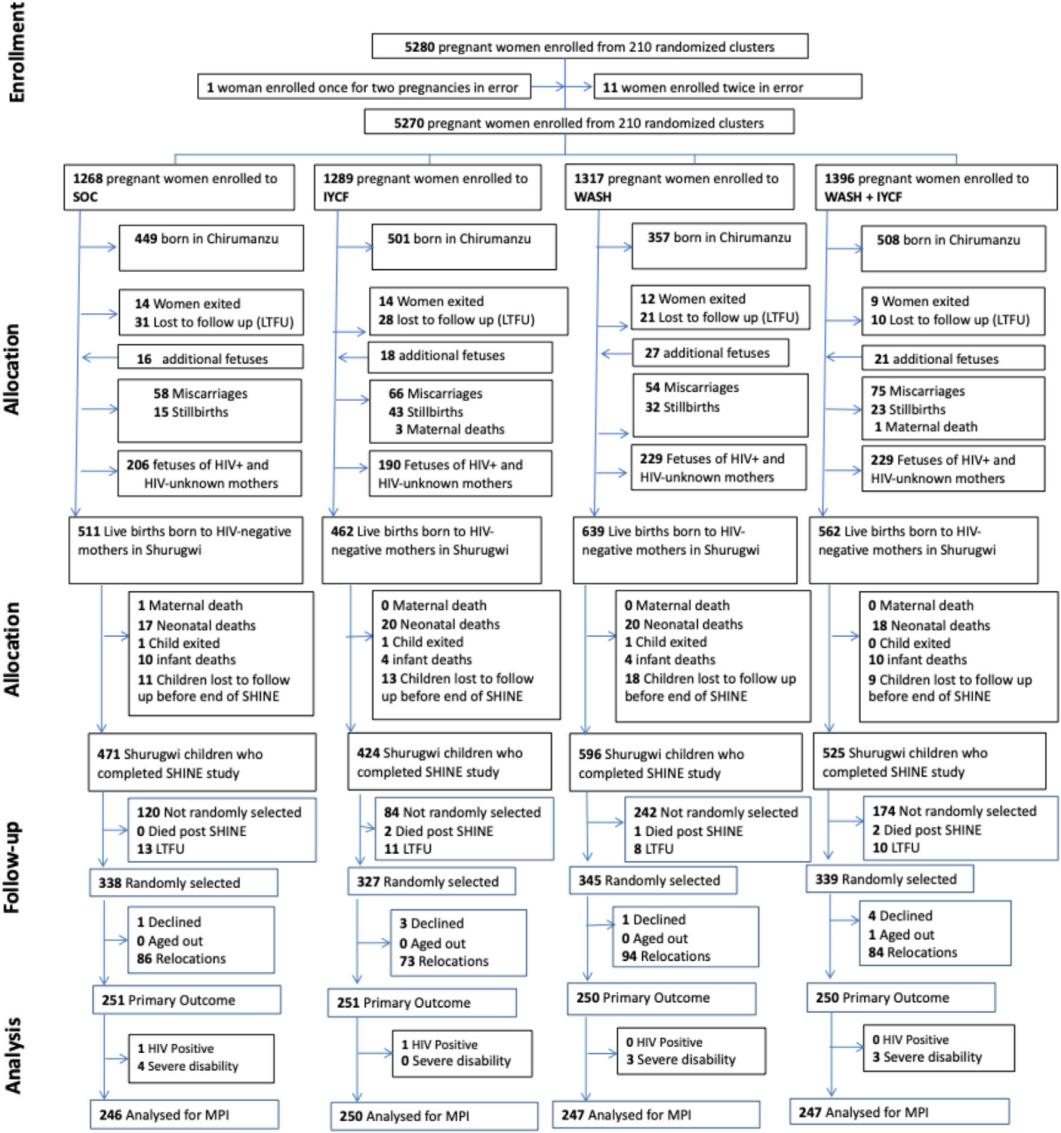
Trial flow. SOC, Standard of care arm; IYCF, Infant and Young Child Feeding intervention arm; WASH, Water, sanitation and hygiene intervention arm; WASH + IYCF: combined arm.

Between 22 November 2012 and 27 March 2015, 5280 pregnant women were enrolled from 211 clusters at median 12 (IQR 9, 16) gestational weeks in Chirumanzu and Shurugwi districts (Fig. 1). Of 2174 HIV-unexposed live births in Shurugwi, 103 (4.7%) children died, 3 (0.1%) voluntarily left the trial, and 51 (2.3%) were lost to follow-up or moved outside Zimbabwe; 2017 children in Shurugwi were therefore assessed at age 18 months.^14^ Five children (0.2%) died and 42 (2.1%) were lost to follow-up after 18 months; 1349 of 1970 available children (68.5%) were randomly selected at age 7 years. Of these, 9 (0.7%) declined follow-up, 337 (25%) had relocated out of Shurugwi, and one child could not be measured before 8 years of age due to heavy rains. In total, 1002 children were assessed at age 7 years between 1st April 2021 and 30th September 2022, including nine sets of twins. Twelve children were excluded from the current analysis due to HIV (2 children) or severe disability (10 children) (Fig. 1). Overall, 990 children were therefore included in the analysis of the primary outcome (246 SOC, 250 IYCF, 247 WASH, 247 IYCF + WASH). 3 further children were unable to perform the finger-tapping test. In the analysis of physical function, 3 children were removed from the shuttle-run test and 1 from the broad jump for medical reasons (e.g. leg injury, asthma) (see Appendix).

Characteristics of mothers and children who were enrolled or not enrolled in the follow-up study were broadly similar (Appendix Table S1), except for more progressive gender norms attitudes and a marginally higher pregnancy MUAC among enrolled women, and higher rates of institutional delivery and weight-for-height Z-score at 18 months among enrolled children. Among household characteristics, there were minor differences in volume of water collected, proportion with an improved water source, livestock inside the house, and food insecurity.

### Follow-up cohort characteristics

Among 1002 children in the long-term follow-up study, 51% were female, with mean (SD) age of 7.3 (0.2) years, mean schooling of 3.2 (0.8) years, height-for-age Z-score of −0.5 (0.9), and weight-for-age Z-score of −0.6 (0.9). Baseline characteristics of those in the follow-up study, measured at enrolment to the original trial during pregnancy, were broadly similar between randomized arms (Table 1).

**Table 1:** Baseline characteristics of mother and children in the SHINE Follow-up study.

	Baseline characteristic	SOC (n = 251)	IYCF (n = 251)	WASH (n = 250)	WASH + IYCF (n = 250)
**Maternal characteristics**	Age, years	25.3 (6.3)	25.5 (6.0)	25.8 (6.8)	26.2 (5.9)
	Maternal height, cm	159.9 (5.9)	160.1 (6.4)	159.6 (5.6)	159.9 (5.9)
	Maternal MUAC, cm	26.2 (3.1)	26.6 (3.2)	26.8 (3.6)	26.7 (3.1)
	Maternal Schooling, years	9.7 (1.8)	9.9 (1.6)	9.6 (1.6)	9.6 (1.7)
	Parity; median (IQR)	2.0 (1.0; 3.0)	1.0 (1.0; 2.0)	1.0 (1.0; 3.0)	2.0 (1.0; 3.0)
	Married	226/240 (94.2%)	219/232 (94.4%)	220/231 (95.2%)	228/236 (96.6%)
	Employed	10/223 (4.5%)	20/222 (9.0%)	24/238 (10.1%)	16/237 (6.8%)
	Religion				
	Apostolic	123/241 (51.0%)	111/233 (47.6%)	112/234 (47.9%)	110/237 (46.4%)
	Christian	101/241 (41.9%)	107/233 (45.9%)	90/234 (38.5%)	100/237 (42.2%)
	Other religion	17/241 (7.1%)	15/233 (6.4%)	32/234 (13.7%)	27/237 (11.4%)
	Gender norm attitudes; median (IQR)	2.7 (1.7; 3.2)	2.7 (1.7; 3.2)	1.7 (1.5; 3.0)	2.0 (1.5; 3.0)
	Perceived social support; median (IQR)	3.5 (3.1; 3.9)	3.7 (3.1; 4.1)	3.6 (3.2; 3.1)	3.7 (3.2; 4.0)
**Household characteristics**	Size, median (IQR)	5.0 (3.0; 6.0)	5.0 (4.0; 7.0)	5.0 (3.0; 6.0)	5.0 (4.0; 6.0)
	Wealth quintiles				
	First (lowest)	48/223 (21.5%)	34/222 (15.3%)	46/237 (19.4%)	37/236 (15.7%)
	Second	40/223 (17.9%)	35/222 (15.8%)	43/237 (18.1%)	50/236 (21.2%)
	Third	41/223 (18.4%)	58/222 (26.1%)	48/237 (20.3%)	40/236 (17.0%)
	Fourth	40/223 (17.9%)	46/222 (20.7%)	52/237 (21.9%)	62/236 (26.3%)
	Fifth (highest)	54/223 (24.2%)	49/222 (22.1%)	48/237 (20.3%)	47/236 (19.9%)
	Electricity to home	7/222 (3.2%)	11/221 (5.0%)	9/238 (3.8%)	4/237 (1.7%)
Other electricity	Generator	8/222 (3.6%)	9/221 (4.1%)	6/238 (2.5%)	9/237 (3.8%)
	Solar	148/222 (66.7%)	156/221 (70.6%)	165/238 (69.3%)	165/237 (69.6%)
	Inverter	4/222 (1.8%)	3/221 (1.4%)	4/238 (1.7%)	2/237 (0.8%)
	no other type	62/222 (27.9%)	53/221 (24.0%)	63/238 (26.5%)	61/237 (25.7%)
Sanitation	Any latrine	73/222 (32.9%)	88/220 (40.0%)	96/234 (41.0%)	88/227 (38.8%)
	Improved latrine	60/222 (27.0%)	73/220 (33.2%)	83/233 (35.6%)	75/227 (33.0%)
Water	Main source of household drinking water is improved	148/222 (66.7%)	155/220 (70.5%)	154/233 (66.1%)	163/229 (71.2%)
	Treat drinking water to make it safer	39/221 (17.6%)	35/219 (16.0%)	36/232 (15.5%)	19/227 (8.3%)
	1 way walk time to fetch water, mins; median (IQR)	10.0 (5.0; 20.0)	10.0 (5.0; 20.0)	10.0 (5.0; 20.0)	10.0 (5.0; 20.0)
	Per capita water volume, L; median (IQR)	6.7 (4.2; 10.0)	6.7 (4.0; 10.0)	6.7 (5.0; 10.0)	6.7 (4.4; 10.0)
Hygiene	Handwashing station with water	16/216 (7.4%)	11/214 (5.1%)	36/229 (15.7%)	38/221 (17.2%)
	Improved floor	116/217 (53.5%)	115/220 (52.3%)	137/233 (58.8%)	119/234 (50.9%)
	Own chickens	177/224 (79.0%)	187/222 (84.2%)	190/236 (80.5%)	199/237 (84.0%)
	Faeces observed in yard	75/239 (31.4%)	92/242 (38.0%)	87/240 (36.3%)	69/241 (28.6%)
Diet quality and food security	Household meets minimum dietary diversity score	76/213 (35.7%)	87/214 (40.7%)	83/227 (36.6%)	85/230 (37.0%)
	Coping Strategies index; median (IQR)	1.0 (0.0; 6.0)	0.0 (0.0; 5.0)	0.0 (0.0; 5.0)	1.0 (0.0; 6.0)
**Child characteristics**	Female	120/251 (47.8%)	121/251 (48.2%)	141/250 (56.4%)	129/250 (51.6%)
	Birthweight, kg	3.1 (0.5)	3.1 (0.5)	3.1 (0.5)	3.1 (0.4)
	Low birthweight (<2.5 kg)	25/237 (10.6%)	20/240 (8.3%)	28/242 (11.6%)	17/238 (7.1%)
	Institutional delivery	213/238 (89.5%)	215/232 (92.7%)	211/235 (89.8%)	216/230 (93.9%)
	Vaginal delivery	235/246 (95.5%)	224/236 (94.9%)	231/245 (94.3%)	219/238 (92.0%)
Trial outcome at 18 months	LAZ at 18 months	−1.6 (1.0)	−1.4 (1.1)	−1.6 (1.0)	−1.5 (1.0)
	Stunted at 18 months	87/249 (34.9%)	61/249 (24.5%)	80/247 (32.4%)	70/248 (28.2%)
	WAZ at 18 months	−0.8 (1.0)	−0.6 (1.0)	−0.8 (1.0)	−0.8 (0.9)
	Head circumference Z-score at 18 months	−0.2 (1.0)	−0.2 (1.0)	−0.3 (1.1)	−0.2 (1.1)
	MUAC Z-score at 18 months	0.2 (0.9)	0.2 (0.9)	−0.01 (0.9)	0.1 (0.8)

n: number, %: percentage, SD, standard deviation; LAZ, length for age Z-score; WAZ, weight-for-age Z-score; MUAC, mid upper arm circumference; IQR, interquartile range. Data are n or n (%), or mean (SD), unless otherwise specified. Maternal and household data were collected approximately 2 weeks after consent was provided (roughly 14 weeks' gestation). This gap resulted in some loss to follow-up between consent and baseline; thus, the number of mothers completing baseline visit is less than the number of mothers with live births. Baseline for infants was at birth. Improved floor defined as concrete, brick, cement or tile; unimproved floor defined as mud, earth, sand, or dung.

### Cognitive outcomes

The effect of the randomised interventions on long-term cognition is shown in Table 2. There was no evidence of an interaction between IYCF and WASH for the primary outcome of mental processing index, and hence for all cognitive outcomes, intervention effects were assessed by combining the two IYCF-containing arms, and combining the two WASH-containing arms. There was no significant effect of IYCF or WASH on the MPI or secondary cognitive outcomes, except for a minor improvement in child socioemotional function in the WASH intervention arms (−0.98 points, 95% CI −1.73, −0.22, p = 0.011) which remained in adjusted analysis.

**Table 2:** Long-term effects of randomised interventions on cognitive function.

Outcome	Effects by arm			Unadjusted					Adjusted model^1^		
	Treatment group	N	Mean (SD)	Treatment Group	N	Mean (SD)	Unadjusted diff (95% CI)	p	n adj	Adjusted diff (95% CI)	p
Mental Processing Index (MPI)	SoC	246	49.2 (12.2)	No IYCF	493	48.8 (11.7)	0.0 (ref)			0.0 (ref)	
	IYCF	250	48.0 (10.4)	IYCF	497	47.8 (10.9)	−0.77 (−2.36, 0.82)	0.344	980	−0.94 (−2.51, 0.63)	0.243
	WASH	247	48.3 (11.1)	No WASH	496	48.6 (11.3)	0.0 (ref)			0.0 (ref)	
	WASH & IYCF	247	47.6 (11.5)	WASH	494	47.9 (11.3)	−0.96 (−2.55, 0.64)	0.239	980	−0.81 (−2.43, 0.81)	0.327
School Achievement Test (SAT)	SoC	246	47.5 (28.5)	No IYCF	493	47.2 (28.2)	0.0 (ref)			0.0 (ref)	
	IYCF	250	46.0 (27.9)	IYCF	497	44.3 (27.4)	−1.74 (−6.42, 2.94)	0.467	980	−1.43 (−5.82, 2.96)	0.524
	WASH	247	46.9 (27.9)	No WASH	496	46.8 (28.2)	0.0 (ref)			0.0 (ref)	
	WASH & IYCF	247	42.5 (26.9)	WASH	494	44.7 (27.4)	−2.34 (−7.02, 2.34)	0.327	980	−1.98 (−6.35, 2.39)	0.375
Executive Function (Plus EF)	SoC	240	114.9 (23.7)	No IYCF	486	113.7 (23.9)	0.0 (ref)			0.0 (ref)	
	IYCF	248	116.0 (23.4)	IYCF	492	115.1 (24.4)	1.49 (−1.75, 4.73)	0.366	968	1.61 (−1.51, 4.73)	0.313
	WASH	246	112.5 (24.2)	No WASH	488	115.5 (23.5)	0.0 (ref)			0.0 (ref)	
	WASH & IYCF	244	114.3 (25.4)	WASH	490	113.4 (24.8)	−2.16 (−5.40, 1.08)	0.192	968	−2.22 (−5.41, 0.97)	0.172
Fine motor, seconds	SoC	244	23.8 (6.5)	No IYCF	491	23.7 (6.2)	0.0 (ref)			0.0 (ref)	
	IYCF	250	24.7 (7.1)	IYCF	495	24.5 (7.0)	0.74 (−0.12, 1.60)	0.091	976	0.73 (−0.11, 1.56)	0.09
	WASH	247	23.6 (6.0)	No WASH	494	24.2 (6.8)	0.0 (ref)			0.0 (ref)	
	WASH & IYCF	245	24.2 (6.8)	WASH	492	23.9 (6.4)	−0.36 (−1.22, 0.50)	0.415	976	−0.63 (−1.51, 0.25)	0.161
Strengths and Difficulties Questionnaire (SDQ)	SoC	245	9.6 (5.3)	No IYCF	492	9.0 (5.3)	0.0 (ref)			0.0 (ref)	
	IYCF	250	8.6 (5.0)	IYCF	497	8.3 (5.0)	−0.70 (−1.46, 0.05)	0.067	979	−0.67 (−1.41, 0.07)	0.076
	WASH	247	8.4 (5.3)	No WASH	495	9.1 (5.1)	0.0 (ref)			0.0 (ref)	
	WASH & IYCF	247	8.0 (5.0)	WASH	494	8.2 (5.2)	−0.98 (−1.73, −0.22)	0.011	979	−0.96 (−1.72, −0.21)	0.013
Child socioem-otional	SoC	242	3.7 (0.7)	No IYCF	485	3.7 (0.7)	0.0 (ref)			0.0 (ref)	
	IYCF	247	3.7 (0.7)	IYCF	488	3.7 (0.7)	0.03 (−0.04, 0.10)	0.42	963	−0.04 (−0.09, 0.01)	0.161
	WASH	243	3.6 (0.8)	No WASH	489	3.7 (0.7)	0.0 (ref)			0.0 (ref)	
	WASH & IYCF	241	3.7 (0.7)	WASH	484	3.7 (0.7)	−0.03 (−0.10, 0.04)	0.40	963	−0.05 (−0.12, 0.01)	0.116

n: number of participants in unadjusted model, n adj: number of participants in adjusted model, SD: standard deviation, 95% CI: 95% confidence interval, unadjusted diff: unadjusted difference, adjusted diff: adjusted difference, Soc: standard of care arm, IYCF: Infant and young child feeding intervention arm, WASH: Water, sanitation and hygiene intervention arm, WASH & IYCF: Combined WASH and IYCF intervention arm. The unadjusted model had no covariates. Adjusted models included study nurse, date measured, exact age of child, ambient temperature, sex of child, maternal depression score (Edinburgh Postnatal Depression Score), household dietary score, maternal dietary score, socioeconomic status as measured by wealth index, birthweight, maternal gender norms, maternal schooling in years, and parity.

### Physical function

The effect of the randomised interventions on physical function is shown in Table 3. There was evidence of an interaction between IYCF and WASH for the outcome of handgrip strength, hence all physical function results were analysed by individual trial arm. IYCF led to increased handgrip strength (0.28 Kg, 95% CI 0.02, 0.53, p = 0.032), compared to standard-of-care, which remained in adjusted analysis. Children in the WASH arm had lower diastolic (−1.75 mm Hg, 95% CI −2.86, −0.65, p = 0.002) and systolic blood pressure (−1.50 mm Hg 95% CI −2.67, −0.33) compared to children in the standard-of-care arm, in adjusted analyses.

**Table 3:** Long-term effects of randomised interventions on physical function.

Outcome	Effects by arm			Unadjusted Model		Adjusted model^1^		
	Treatment group	n	Mean (SD)	Unadjusted diff (95% CI)	p	n adj	Adjusted diff (95% CI)	p
Grip strength, kg	SoC	246	10.6 (1.8)	0.0 (ref)			0.0 (ref)	
	IYCF	250	10.8 (2.0)	0.28 (0.02, 0.53)	0.032		0.22 (−0.01, 0.45)	0.056
	WASH	247	10.7 (2.0)	0.13 (−0.19, 0.44)	0.421	980	0.04 (−0.28, 0.36)	0.8
	WASH & IYCF	247	10.6 (1.9)	0.05 (−0.23, 0.33)	0.734		0.06 (−0.17, 0.29)	0.616
Broad jump, cm	SoC	245	112.8 (15.0)	0.0 (ref)			0.0 (ref)	
	IYCF	249	112.2 (16.4)	−0.60 (−3.45, 2.26)	0.683		−0.92 (−3.69, 1.86)	0.517
	WASH	246	113.0 (14.1)	0.14 (−2.70, 2.98)	0.922	977	0.01 (−2.88, 2.91)	0.993
	WASH & IYCF	247	113.0 (15.0)	0.19 (−2.74, 3.13)	0.899		−0.24 (−3.24, 2.76)	0.875
VO2 max (Shuttle run test), ml/kg/min	SoC	245	51.1 (2.7)	0.0 (ref)			0.0 (ref)	
	IYCF	248	50.8 (2.5)	−0.37 (−0.91, 0.17)	0.183		−0.43 (−0.94, 0.09)	0.104
	WASH	247	50.6 (3.0)	−0.49 (−1.07, 0.09)	0.098	975	−0.26 (−0.84, 0.32)	0.38
	WASH & IYCF	246	50.9 (2.6)	−0.14 (−0.64, 0.36)	0.575		−0.08 (−0.58, 0.42)	0.758
Resting Diastolic Blood Pressure, mm Hg	SoC	245	62.4 (7.5)	0.0 (ref)			0.0 (ref)	
	IYCF	250	62.4 (7.8)	−0.20 (−1.27, 0.88)	0.717		−0.26 (−1.16, 0.63)	0.563
	WASH	246	61.7 (7.1)	−0.85 (−1.92, 0.21)	0.115	978	−1.75 (−2.86, −0.65)	0.002
	WASH & IYCF	247	62.8 (7.6)	0.54 (−0.65, 1.72)	0.374		−0.1 (−1, 0.8)	0.826
Resting systolic Blood Pressure, mm Hg	SoC	245	97.1 (9.2)	0.0 (ref)			0.0 (ref)	
	IYCF	250	96.8 (9.1)	−0.42 (−2.01, 1.16)	0.599		−0.36 (−1.65, 0.93)	0.584
	WASH	246	96.4 (9.0)	−0.78 (−2.10, 0.55)	0.252	978	−1.5 (−2.67, −0.33)	0.012
	WASH & IYCF	247	97.7 (9.8)	0.53 (−1.01, 2.07)	0.503		−0.17 (−1.7, 1.37)	0.829

n: number of participants in unadjusted model, n adj: number of participants in adjusted model, SD: standard deviation, 95% CI: 95% confidence interval, unadjusted diff: unadjusted difference, adjusted diff: adjusted difference, Soc: standard of care arm, IYCF: Infant and young child feeding intervention arm, WASH: Water, sanitation and hygiene intervention arm, WASH& IYCF: Combined WASH and IYCF intervention arm. VO_2_max: maximum aerobic capacity derived from level achieved in the 20m shuttle run test. The unadjusted model had no covariates. Adjusted models included study nurse, date measured, exact age of child, ambient temperature, sex of child, maternal depression score (Edinburgh Postnatal Depression Score), household dietary score, maternal dietary score, socioeconomic status as measured by wealth index, birthweight, maternal gender norms, maternal schooling in years, and parity.

### Growth and body composition

The effect of the randomised interventions on growth and body composition is shown in Table 4. There was no evidence of an interaction between the IYCF and WASH interventions on height-for-age Z-score, and hence for all growth and body composition outcomes, the effects of the interventions were assessed by combining the two IYCF-containing arms, and similarly combining the two WASH-containing arms. There were no significant effects of the IYCF or WASH interventions on any growth or body composition measures at age 7 years.

**Table 4:** Long-term effects of randomised interventions on growth and body composition.

Outcome	Effects by arm			Unadjusted model					Adjusted model		
	Treatment group	N	Mean (SD)	Treatment group	N	Mean S(D)	Unadjusted diff (95% CI)	p	N adj	Adjusted diff (95% CI)	p
HAZ	SoC	246	−0.6 (0.8)	No IYCF	493	−0.5 (0.8)	0.0 (ref)			0.0 (ref)	
	IYCF	250	−0.4 (0.9)	IYCF	497	−0.5 (0.9)	0.09 (−0.01, 0.18)	0.093	980	0.06 (−0.04, 0.16)	0.226
	WASH	247	−0.5 (0.8)	No WASH	496	−0.5 (0.9)	0.0 (ref)			0.0 (ref)	
	WASH & IYCF	247	−0.5 (0.9)	WASH	494	−0.5 (0.9)	0.02 (−0.08, 0.12)	0.711	980	0.02 (−0.08, 0.12)	0.759
WAZ	SoC	245	−0.7 (0.9)	No IYCF	492	−0.7 (0.9)	0.0 (ref)			0.0 (ref)	
	IYCF	249	−0.6 (0.9)	IYCF	496	−0.6 (0.8)	0.04 (−0.06, 0.14)	0.447	978	0.02 (−0.08, 0.12)	0.683
	WASH	247	−0.6 (0.9)	No WASH	494	−0.6 (0.9)	0.0 (ref)			0.0 (ref)	
	WASH & IYCF	247	−0.7 (0.8)	WASH	494	−0.6 (0.8)	0.01 (−0.10, 0.11)	0.898	978	0.00 (−0.11, 0.10)	0.979
BMIZ	SoC	245	−0.5 (0.9)	No IYCF	492	−0.5 (0.9)	0.0 (ref)			0.0 (ref)	
	IYCF	249	−0.5 (0.8)	IYCF	496	−0.5 (0.8)	−0.04 (−0.16, 0.08)	0.538	978	−0.04 (−0.16, 0.08)	0.490
	WASH	247	−0.5 (0.9)	No WASH	494	−0.5 (0.8)	0.0 (ref)			0.0 (ref)	
	WASH & IYCF	247	−0.6 (0.8)	WASH	494	−0.5 (0.9)	−0.04 (−0.16, 0.08)	0.541	978	−0.05 (−0.18, 0.08)	0.425
Knee-heel length, cm	SoC	246	37.2 (1.9)	No IYCF	492	37.3 (1.9)	0.0 (ref)			0.0 (ref)	
	IYCF	250	37.6 (1.9)	IYCF	497	37.5 (2.0)	0.16 (−0.06, 0.37)	0.152	979	0.14 (−0.07, 0.34)	0.194
	WASH	246	37.4 (1.9)	No WASH	496	37.4 (1.9)	0.0 (ref)			0.0 (ref)	
	WASH & IYCF	247	37.4 (2.0)	WASH	493	37.4 (1.9)	0.05 (−0.17, 0.27)	0.655	979	0.03 (−0.19, 0.24)	0.797
Head circumference, cm	SoC	246	51.3 (1.4)	No IYCF	493	51.2 (1.4)	0.0 (ref)			0.0 (ref)	
	IYCF	250	51.4 (1.5)	IYCF	497	51.4 (1.4)	0.11 (−0.03, 0.26)	0.122	980	0.05 (−0.08, 0.17)	0.451
	WASH	247	51.2 (1.5)	No WASH	496	51.3 (1.4)	0.0 (ref)			0.0 (ref)	
	WASH & IYCF	247	51.4 (1.3)	WASH	494	51.3 (1.4)	−0.01 (−0.16, 0.13)	0.843	980	0.09 (−0.06, 0.24)	0.231
Mid-upper arm circumference (MUAC), cm	SoC	246	16.8 (1.3)	No IYCF	493	16.9 (1.3)	0.0 (ref)			0.0 (ref)	
	IYCF	249	17.0 (1.3)	IYCF	496	17.0 (1.3)	0.10 (−0.08, 0.28)	0.288	979	0.08 (−0.09, 0.26)	0.343
	WASH	247	16.9 (1.3)	No WASH	495	16.9 (1.3)	0.0 (ref)			0.0 (ref)	
	WASH & IYCF	247	16.9 (1.3)	WASH	494	16.9 (1.3)	0.01 (−0.17, 0.19)	0.926	979	−0.05 (−0.22, 0.12)	0.576
Waist circumference, cm	SoC	246	54.0 (3.1)	No IYCF	493	54.1 (3.2)	0.0 (ref)			0.0 (ref)	
	IYCF	249	54.2 (3.1)	IYCF	496	54.1 (3.0)	−0.01 (−0.41, 0.39)	0.957	979	−0.04 (−0.44, 0.36)	0.838
	WASH	247	54.1 (3.4)	No WASH	495	54.1 (3.1)	0.0 (ref)			0.0 (ref)	
	WASH & IYCF	247	53.9 (2.9)	WASH	494	54.0 (3.2)	−0.09 (−0.49, 0.30)	0.643	979	−0.12 (−0.55, 0.31)	0.59
Hip circumference, cm	SoC	246	60.7 (3.8)	No IYCF	493	60.8 (4)	0.0 (ref)			0.0 (ref)	
	IYCF	250	61.2 (3.9)	IYCF	497	60.9 (3.9)	0.08 (−0.46, 0.63)	0.765	980	0.06 (−0.45, 0.57)	0.818
	WASH	247	61.0 (4.1)	No WASH	496	60.9 (3.9)	0.0 (ref)			0.0 (ref)	
	WASH & IYCF	247	60.7 (3.8)	WASH	494	60.8 (4.0)	−0.13 (−0.67, 0.42)	0.652	980	−0.21 (−0.76, 0.34)	0.455
Calf circumference, cm	SoC	245	23.3 (1.7)	No IYCF	492	23.4 (1.7)	0.0 (ref)			0.0 (ref)	
	IYCF	250	23.5 (1.7)	IYCF	497	23.5 (1.6)	0.11 (−0.10, 0.33)	0.299	979	0.08 (−0.12, 0.28)	0.418
	WASH	247	23.4 (1.7)	No WASH	495	23.4 (1.7)	0.0 (ref)			0.0 (ref)	
	WASH & IYCF	247	23.4 (1.6)	WASH	494	23.4 (1.7)	0.01 (−0.20, 0.23)	0.907	979	−0.07 (−0.28, 0.15)	0.541
Lean Mass Index, Ohms^−1^	SoC	243	12.0 (1.3)	No IYCF	488	12.1 (1.4)	0.0 (ref)			0.0 (ref)	
	IYCF	248	12.1 (1.3)	IYCF	494	12.2 (1.2)	0.09 (−0.08, 0.26)	0.310	972	−0.04 (−0.18, 0.1)	0.608
	WASH	245	12.1 (1.4)	No WASH	491	12.1 (1.3)	0.0 (ref)			0.0 (ref)	
	WASH & IYCF	246	12.2 (1.2)	WASH	491	12.2 (1.3)	0.08 (−0.09, 0.25)	0.360	972	0.07 (−0.07, 0.22)	0.327
Impedance Index M^2^ Ohms^−1^	SoC	243	1.7 (0.3)	No IYCF	488	1.7 (0.3)	0.0 (ref)			0.0 (ref)	
	IYCF	248	1.8 (0.3)	IYCF	494	1.8 (0.2)	0.02 (−0.01, 0.05)	0.138	972	0.00 (−0.02, 0.03)	0.834
	WASH	245	1.8 (0.3)	No WASH	491	1.7 (0.3)	0.0 (ref)			0.0 (ref)	
	WASH & IYCF	246	1.8 (0.2)	WASH	491	1.8 (0.3)	0.02 (−0.01, 0.05)	0.262	972	0.02 (−0.01, 0.04)	0.238
Phase Angle, ^0^	SoC	243	5.0 (0.6)	No IYCF	489	4.9 (0.6)	0.0 (ref)			0.0 (ref)	
	IYCF	247	4.9 (0.6)	IYCF	493	4.9 (0.5)	0.00 (−0.07, 0.07)	0.906	972	0.01 (−0.06, 0.08)	0.812
	WASH	246	4.9 (0.5)	No WASH	490	5.0 (0.6)	0.0 (ref)			0.0 (ref)	
	WASH & IYCF	246	4.9 (0.5)	WASH	492	4.9 (0.5)	−0.03 (−0.10, 0.04)	0.418	972	−0.04 (−0.1, 0.02)	0.172
Total skinfold thickness, mm	SoC	245	26.8 (6.1)	No IYCF	492	27.1 (6.4)	0.0 (ref)			0.0 (ref)	
	IYCF	249	27.4 (5.8)	IYCF	495	27.0 (5.9)	−0.02 (−0.86, 0.82)	0.963	978	0.00 (−0.77, 0.78)	0.997
	WASH	247	27.3 (6.8)	No WASH	494	27.1 (5.9)	0.0 (ref)			0.0 (ref)	
	WASH & IYCF	246	26.6 (6.1)	WASH	493	27.0 (6.4)	−0.19 (−1.03, 0.66)	0.666	978	−0.45 (−1.23, 0.32)	0.254
Peripheral skinfold thickness, mm	SoC	245	16.1 (3.7)	No IYCF	492	16.2 (3.8)	0.0 (ref)			0.0 (ref)	
	IYCF	249	16.6 (3.7)	IYCF	496	16.2 (3.7)	0.03 (−0.47, 0.53)	0.913	978	−0.02 (−0.48, 0.44)	0.944
	WASH	247	16.2 (3.9)	No WASH	494	16.3 (3.7)	0.0 (ref)			0.0 (ref)	
	WASH & IYCF	247	15.8 (3.6)	WASH	494	16.0 (3.7)	−0.37 (−0.87, 0.13)	0.143	978	−0.47 (−0.95, 0.01)	0.053
Central skinfold thickness, mm	SoC	246	10.8 (3.4)	No IYCF	493	11 (3.3)	0.0 (ref)			0.0 (ref)	
	IYCF	250	10.9 (2.7)	IYCF	496	10.9 (2.8)	−0.08 (−0.52, 0.36)	0.718	980	−0.02 (−0.44, 0.39)	0.915
	WASH	247	11.1 (3.3)	No WASH	496	10.9 (3.0)	0.0 (ref)				
	WASH & IYCF	246	10.9 (3.0)	WASH	493	11 (3.1)	0.10 (−0.35, 0.54)	0.671	980	−0.12 (−0.54, 0.3)	0.578
Haemoglobin g/dl	SoC	246	12.7 (1.3)	No IYCF	493	12.7 (1.2)	0.0 (ref)			0.0 (ref)	
	IYCF	250	12.6 (1.1)	IYCF	497	12.6 (1.1)	−0.04 (−0.20, 0.12)	0.624	980	−0.04 (−0.2, 0.13)	0.656
	WASH	247	12.7 (1.2)	No WASH	496	12.7 (1.2)	0.0 (ref)				
	WASH & IYCF	247	12.7 (1.2)	WASH	494	12.7 (1.2)	−0.01 (−0.17, 0.15)	0.865	980	0.01 (−0.15, 0.17)	0.911

n: number of participants in unadjusted model, n adj: number of participants in adjusted model, SD: standard deviation, 95% CI: 95% confidence interval, unadjusted diff: unadjusted difference, adjusted diff: adjusted difference, Soc: standard of care arm, IYCF: Infant and young child feeding intervention arm, WASH: Water, sanitation and hygiene intervention arm, WASH& IYCF: Combined WASH and IYCF intervention arm. HAZ: Height-for-age Z-score at 7 years, WAZ: Weight-for-age Z-score at 7 years, BMIZ: BMI-for-age Z-score at 7 years. The unadjusted model had no covariates. Adjusted models included study nurse, date measured, exact age of child, ambient temperature, sex of child, maternal depression score (Edinburgh Postnatal Depression Score), household dietary score, maternal dietary score, socioeconomic status as measured by wealth index, birthweight, maternal gender norms, maternal schooling in years, and parity.

### Sensitivity analysis

There was evidence of interaction (p < 0.05) between child sex and trial intervention arm for two outcomes (Supporting Information). Boys receiving IYCF had better grip strength than boys in the SOC arm (0.53 Kg, 95% CI 0.19, 0.87 p = 0.002), whilst girls showed no difference between arms. For VO_2_ max, girls appeared to benefit in the combined IYCF + WASH arm when compared to the SOC arm (0.58, 95% CI 0.07, 1.08, p = 0.025).

## Discussion

We investigated the long-term effects of improved WASH and improved IYCF on school-age growth, physical and cognitive function in a setting of high stunting prevalence in rural Zimbabwe. We had previously shown that IYCF, which comprised complementary feeding education and small-quantity lipid-based nutrient supplements, modestly reduced stunting and anaemia at age 18 months, while WASH had no effects in early life. Our goal was to explore whether these interventions had any long-term functional benefits for children at age 7 years. We found minimal evidence that either WASH or IYCF improved long-term child cognitive, physical or growth outcomes. There is therefore a need to consider more comprehensive packages of nurturing care, which may need to start earlier and be delivered for longer, to enhance human capital across the life-course.

Very few studies have measured long-term physical or cognitive function after IYCF interventions. Data from the landmark INCAP study in Guatemala 50 years ago indicated that early-life improvements in nutrition could confer long-term benefits for cognition^38^; however, this study was conducted at a time when global stunting prevalence was much higher,^2^ and the intervention was started earlier (during pregnancy for some participants) and had a larger impact on linear growth (+0.62 HAZ)^38^ than the current study. A small global effect on cognition was reported in Jamaica at age 6 years using milk-based formula,^39^ but this was not sustained by 17–18 years.^40^ IYCF is still considered the most effective intervention to prevent stunting during the vulnerable window between 6 and 18 months of age,^4^ and recent meta-analyses show that SQ-LNS modestly increases linear growth (average + 0.11 HAZ)^5^ and early child development (approximately 0.11–0.13 standardised effect size in populations with a high prevalence of stunting).^9^ The (iLiNS)-DYAD study in Ghana showed an improvement of 0.16 Z-scores in socioemotional behaviour for children aged 5 years randomised to SQ-LNS.^16^ Despite clear short-term benefits of SQ-LNS, there is limited evidence as to whether early-life gains in growth and cognition provide tangible benefits as children mature. In this study, we show no long-term effects of SQ-LNS on cognitive function, using a detailed battery of tests which were adapted for use in rural Zimbabwe.^17^ This may be expected as we did not find effects of SQ-LNS on neurodevelopment in this cohort at age 2 years^14^; however, we had hoped that effects may be apparent in more detailed assessments at older ages. Children who received the WASH intervention had no clear long-term benefits except for a marginal improvement in the caregiver-reported child socioemotional function—a cognitive domain that could only be measured at school age. This potential benefit of WASH has been previously postulated,^41^ although few studies have measured socioemotional outcomes. Community health workers performed the same number of visits in control and intervention arms so this could not be explained by more contact time with the CHW. Given the similar baseline characteristics between those enrolled or not enrolled in the follow-up study (Appendix Table S1), we believe this can be considered a representative cohort for the generalisability of the study findings.

As we found improvements in linear growth in the IYCF arm at 18 months of age, we anticipated that long-term effects on physical function were plausible. We found modest gains in hand grip strength following IYCF. This was seen only in boys, plausibly because they are more biologically vulnerable to adverse conditions and more responsive to early-life interventions.^42^ However, there were no other long-term increases in growth, lean mass, or broad jump; therefore the mechanism of intervention effect is unclear, unless muscle quality was improved due to the high-quality protein in SQ-LNS. However, given the large number of outcome measures, it is also possible that this was due to type 1 error. Further studies are needed since long-term improvements in muscle growth, strength and quality would be an important finding for lifelong health, because muscle mass tracks into adult life and sarcopenia predicts all-cause mortality during ageing.^43^

There are three potential explanations for our finding of minimal functional benefits by school-age following early-life interventions. First, there may genuinely be no long-term effect of SQ-LNS, if benefits of the nutrition intervention are only found during the period of supplementation, or catch-up growth in the other trial arms negated any early benefits in the nutrition arms.^44^ For the WASH intervention, we anticipated no long-term benefits since we found no impact on growth at 18 months^13^ or on cognitive development at 24 months of age.^14^ Second, there may be a long-term effect of early-life interventions, but we failed to detect differences due to our choice of tests, the study power, or sampling strategy. However the test battery was extensively piloted,^17^ the sample size was designed to detect plausible effect sizes for cognition (assuming no interactions between interventions), and there were no major baseline differences suggesting a bias in enrolment to this follow-up study. Nevertheless, it remains possible that we missed a small effect due to the nutrition intervention.^9^ Third, certain children may have had long-term benefits from the IYCF intervention, but not all had the potential to respond in a sustained manner.^45^ It is plausible that multiple adversities, socioeconomic deprivation, and inadequate nurturing care may hinder or outweigh any of the initial benefits. As has previously been posited, the child's environment may be the primary driver of both poor functional development and poor growth,^46^ rather than growth being a reliable proxy measure for function. Recent studies from Brazil have shown reductions in stunting following national socioeconomic development and equity-based policies.^47^

This study has several strengths. We re-enrolled a large cohort of children several years after the end of the SHINE trial and conducted a holistic battery of tests to evaluate long-term effects of multimodal early-life interventions. Our choice of tests was informed by the best available assessment tools which we carefully piloted and refined in rural Zimbabwe.^17^^,^^26^ Furthermore, study nurses were extensively trained, monitored and standardised. This study also has some limitations. Firstly, we only re-enrolled children from one of the original two SHINE districts due to cost and logistical considerations, plus there was outward migration from the study area. However, comparisons of baseline characteristics between those enrolled and not enrolled, and between trial intervention arms for those enrolled, showed no major differences that we believe would bias our results. Our sample size enabled us to detect a 0.2 standard deviation difference in the mental processing index between intervention and control arms, which is similar to the magnitude of difference previously reported for other effective interventions in early child development such as early-life stimulation,^48^^,^^49^ parenting interventions,^50^ and conditional cash transfers.^51^ Finally, our comprehensive battery of tests increases our confidence that there is no consistent pattern of long-term functional gains from either intervention. The large number of secondary outcomes, and the fact that the study was underpowered to test for interactions, increases the chance that the few benefits we observed are due to statistical type 1 error, and further studies are therefore needed to confirm or refute these findings.

In summary, we report the first long-term follow-up of a cluster-randomised trial of early-life nutrition in sub-Saharan Africa, which measured body composition, cognitive and physical function. The minimal effects of IYCF or WASH interventions by school-age, despite modest early-life growth benefits following SQ-LNS, calls for further research into interventions that can sustainably improve long-term cognition and physical function. Our findings do not challenge the clear benefits of SQ-LNS for short-term growth, survival and early child development.^6^^,^^9^^,^^52^ However, recent meta-analyses have shown that nurturing care and parenting interventions may be needed to have substantial effects on child cognitive development.^10^^,^^50^^,^^53^^,^^54^ Therefore, interventions that start earlier, are provided for longer, and promote more holistic nurturing care, may be more effective at transforming the child's environment and driving long-term benefits for child function.

## Contributors

JDP, RN and AJP were responsible for conceptualisation, data curation, formal analysis, funding acquisition, investigation, methodology, project administration, resources, software, supervision, validation, visualisation, writing—original draft, writing—review & editing. CM was responsible for conceptualisation, data curation, investigation, methodology, project administration, resources, supervision, validation, writing—review & editing. MMwapaura was responsible for conceptualisation, data curation, formal analysis, investigation, methodology, project administration, resources, software, supervision, validation, writing—review & editing.

GM, IM, TMashedze, EMunyama were responsible for conceptualisation, data curation, investigation, methodology, validation, writing—review & editing. MK, TMashiri, KS, DM, MT, SN, AS, MMangwende were responsible for data curation, investigation, methodology, writing—review & editing. GM was responsible for data curation, formal analysis, investigation, project administration, software, supervision, validation, writing—review & editing. DC was responsible for conceptualisation, data curation, investigation, methodology, project administration, resources, supervision, validation, writing—review & editing. EMpofu was responsible for data curation, formal analysis, investigation, software, writing—review & editing. JT was responsible for data curation, formal analysis, investigation, software, writing—review & editing, BM and BC were responsible for data curation, formal analysis, investigation, supervision, software, writing—review & editing, CN, MM, HN, VS, MJG, JCW and EA were responsible for conceptualisation, investigation, methodology, project administration, writing—review & editing, LHM, MS and JH were responsible for conceptualisation, investigation, methodology, formal analysis, project administration, writing—review & editing, LFL and NVT were responsible for conceptualisation, investigation, methodology, project administration, writing—review & editing, JDP, JT and RN had access to and verified all the data. All authors read and approved the manuscript.

## Data sharing statement

Data will be freely available as individual participant data on ClinEpiDB with an accompanying data dictionary at http://ClinEpiDB.org from 2025. Researchers must agree to the policies and comply with the mechanism of ClinepiDB to access datahoused on this platform. Prior to that time, data are available upon reasonable request from the Zvitambo Institute for Maternal and Child Health Research, by contacting Dr Robert Ntozini (r.ntozini@zvitambo.com).

## Declaration of interests

There are no interests to declare from any authors.

## Appendix A Supplementary data

Supplementary Figure and Tables

CONSORT_ROUTINE_checklist
