# Supplementary material for: School-age growth and development following infant feeding and/or water, sanitation, and hygiene interventions in rural Zimbabwe: long-term follow-up of a cluster-randomised trial
Source: eClinicalMedicine. 2024 Nov 22;78:102946. doi: 10.1016/j.eclinm.2024.102946 (PMC11617972; doi:10.1016/j.eclinm.2024.102946)
Supplement: Supplementary Figure and Tables [file mmc1.docx]

**Supplementary appendix**

Table of Contents

[Extended results 1](#_Toc162435265)

[Table S1: Enrolment in SFU 1](#_Toc162435266)

[Table S2: Baseline Characteristics by arm 3](#_Toc162435267)

[Table S3: effect of intervention by child sex 5](#_Toc162435268)

[Table S4-1 to S4-5: Long-term effects of randomised interventions on Cognition subtests 7](#_Toc162435269)

[Table S5-1 to S5-2: Secondary physical function outcomes 14](#_Toc162435270)

[Table S6: Secondary growth outcomes 17](#_Toc162435271)

[Expanded methods 18](#_Toc162435272)

[Figure S1: Directed acyclic graph for adjusted models 18](#_Toc162435273)

[Validation and quality control of SAHARAN toolbox 18](#_Toc162435274)

[Definition of disability 20](#_Toc162435275)

[List of abbreviations 20](#_Toc162435276)

[References 21](#_Toc162435277)

## Extended results

### Table S1: Enrolment in SFU

| **Supplementary table 1** | **HUU included in SFU (1002)** | **HUU Not included in SFU** | p-value |
| --- | --- | --- | --- |
| Caregiver assessed, N | 1002 | 2987 |  |
| Children assessed (HIV negative), N | 990 | 2949 |  |
| Women completing baseline visit, N | 922 | 2767 |  |
| **Household characteristics** |  |  |  |
| Median number of occupants [IQR] | 5.0 (4.0; 6.0) | 5.5 (3.0 ; 6.0) | 0.086 |
| Wealth quintile, percent [n] |  |  |  |
| Lowest | 164/914 (17.9%) | 511/2744 (18.6%) | 0.837 |
| Second | 168/914 (18.4%) | 544/2744 (19.8%) |  |
| Middle | 186/914 (20.4%) | 553/2744 (20.2%) |  |
| Fourth | 200/914 (21.9%) | 576/2744 (21.0%) |  |
| Highest | 196/914 (21.4%) | 560/2744 (20.4%) |  |
| **Electricity** |  |  |  |
| Electricity, yes n (%) | 31/914 (3.4%) | 69/2738 (2.5%) | 0.179 |
| Other electric power |  |  |  |
| Generator | 32/914 (3.5%) | 86/2743 (3.1%) | 0.205 |
| Solar panel | 631/914 (69.0%) | 1795/2743 (65.4%) |  |
| Inverter | 13/914 (1.4%) | 44/ 2743 (1.6%) |  |
| No other type | 238/914 (26.0%) | 818/2743 (29.8%) |  |
| **Sanitation** |  |  |  |
| Any latrine at household | 343/899 (38.2%) | 981/2707 (36.2%) | 0.419 |
| Improved latrine at household | 290/898 (32.3%) | 867/2703 (32.1%) | 0.924 |
| **Water** |  |  |  |
| Main source of household drinking water improved | 618/900 (68.7%) | 1675/2725 (61.5%) | 0.014 |
| Treat drinking water to make it safer | 127/895 (14.2%) | 322/2673 (12.1%) | 0.120 |
| One-way walk time to fetch drinking water (min) , median (IQR) | 10.0 (5.0 ; 20.0) | 10.0 (5.0 ; 20.0) | 0.302 |
| Per capita water volume collected past 24 hr, median (IQR) | 6.7 (4.4 ; 10.0) | 7.5 (5.0 ; 12.0) | <0.001 |
| **Hygiene** |  |  |  |
| Handwashing station at household | 101/876 (11.5%) | 207/2557 (8.1%) | 0.012 |
| Improved floor | 484/900 (53.8%) | 1512/2707 (55.9%) | 0.441 |
| Number of chickens, median (IQR) | 6.0 (2.0 ; 10.0) | 6.0 (2.0 ; 10.0) | 0.120 |
| Livestock observed inside the house | 390/963 (40.5%) | 1039/2886 (36.0%) | 0.017 |
| Faeces observed in the yard | 322/958 (33.6%) | 877/2879 (30.5%) | 0.146 |
| **Diet quality and food security** |  |  |  |
| Household meets minimum dietary diversity score | 328/880 (37.3% | 965/2344 (41.2%) | 0.095 |
| Coping strategies index, median (IQR) | 0.0 (0.0 ; 6.0) | 1.0 (0.0 ; 7.0) | 0.008 |
| **Maternal characteristics** |  |  |  |
| Mean age (SD), years | 25.7 (6.3) | 25.6 (6.7) | 0.147 |
| Mean height (SD), cm] | 159.9 (6.0) | 160.2 (5.6) | 0.083 |
| Mean mid-upper-arm circumference (SD), cm | 26.6 (3.3) | 26.3 (3.0) | 0.046 |
| Maternal Hb | 12.2 (1.4) | 12.1 (1.5) | 0.207 |
| Mother meets minimum dietary diversity score | 330/901 (36.6%) | 1081/2675 (40.4%) | 0.100 |
| Mean years of schooling completed (SD) | 9.7 (1.7) | 9.6 (1.8) | 0.096 |
| Median parity (IQR) | 2.0 (1.0 ; 3.0) | 2.0 (1.0 ; 3.0) | 0.027 |
| Married | 889/935 (95.1%) | 2657/2782 (95.5%) | 0.289 |
| Employed | 69/916 (7.5%) | 242/2739 (8.8%) | 0.228 |
| Religion |  |  |  |
| Apostolic | 455/941 (48.4%) | 1308/2804 (46.7%) | 0.097 |
| Other Christian | 395/941 (42.0%) | 1289/2804 (46.0%) |  |
| Other religion | 91/941 (9.7%) | 207/2804 (7.4%) |  |
| Maternal capabilities |  |  |  |
| Median Gender norms and attitudes (IQR) | 2.3 (1.5 ; 3.0) | 1.7 (1.5 ; 3.0) | <0.001 |
| Median Perceived social support (IQR) | 3.6 (3.2 ; 4.0) | 3.7 (3.3 ; 4.0) | 0.050 |
| Median EPDS depression scale (IQR) | 1.0 (0.0 ; 5.0) | 1.0 (0.0 ; 4.0) | 0.809 |
| **Infant characteristics** |  |  |  |
| Female, percent | 511/1002 (51.0%) | 1451/2972 (48.8%) | 0.279 |
| Mean birth weight (SD), kg | 3.1 (0.5) | 3.1 (0.5) | 0.434 |
| Low birthweight | 90/957 (9.4%) | 235/2616 (9.0%) | 0.738 |
| Institutional delivery | 855/935 (91.4%) | 2353/2669 (88.2%) | 0.008 |
| Vaginal delivery | 909/965 (94.2%) | 2482/2699 (92.0%) | 0.098 |
| **18 month endpoint characteristics** |  |  |  |
| Mean LAZ at 18 months, (SD) | -1.5 (1.0) | -1.5 (1.1) | 0.175 |
| Mean WAZ at 18 months, (SD) | -0.7 (1.0) | -0.7 (1.0) | 0.055 |
| Mean WHZ at 18 months, (SD) | -0.04 (1.0) | 0.1 (1.1) | 0.001 |
| Mean Head circumference at 18 months, (SD) | -0.2 (1.1) | -0.2 (1.5) | 0.477 |
| Mean MUAC at 18 months, (SD) | 0.1 (0.9) | 0.02 (0.9) | 0.026 |
| Mean Hb at 18 months, (SD) | 11.8 (1.1) | 11.7 (1.2) | 0.115 |

Table S1: HIV exposed uninfected (HUU) children enrolled in SHINE follow-up (SFU) compared to those not enrolled. Household, maternal and infant characteristics are included.

### Table S2: Baseline Characteristics by arm

|  | **Baseline characteristics by arm (SFU) for HUU** | | | | |
| --- | --- | --- | --- | --- | --- |
| **Demographics** | Caregiver assessed at 7 yr | 247 | 250 | 247 | 248 |
|  | Child assessed at 7 yr | 251 | 251 | 250 | 250 |
|  | Women completing baseline visit | 224 | 224 | 240 | 238 |
| **Household characteristics** | Size, median (IQR) | 5.0 (3.0 ; 6.0) | 5.0 (4.0 ; 7.0) | 5.0 (3.0 ; 6.0) | 5.0 (4.0 ; 6.0) |
|  | Wealth quintiles |  |  |  |  |
|  | Lowest | 48/223 (21.5%) | 34/222 (15.3%) | 46/237 (19.4%) | 37/236 (15.7%) |
|  | second | 40/223 (17.9%) | 35/222 (15.8%) | 43/237 (18.1%) | 50/236 (21.2%) |
|  | Third | 41/223 (18.4%) | 58/222 (26.1%) | 48/237 (20.3%) | 40/236 (17.0%) |
|  | Fourth | 40/223 (17.9%) | 46/222 (20.7%) | 52/237 (21.9%) | 62/236 (26.3%) |
|  | Fifth | 54/223 (24.2%) | 49/222 (22.1%) | 48/237 (20.3%) | 47/236 (19.9%) |
|  | Electricity to home | 7/222(3.2%) | 11/221 (5.0%) | 9/238 (3.8%) | 4/237 (1.7%) |
| Other electricity | Generator | 8/222 (3.6%) | 9/221 (4.1%) | 6/238 (2.5%) | 9/237 (3.8%) |
|  | Solar | 148/222 (66.7%) | 156/221 (70.6%) | 165/238 (69.3%) | 165/237 (69.6%) |
|  | Inverter | 4/222 (1.8%) | 3/221 (1.4%) | 4/238 (1.7%) | 2/237 (0.8%) |
|  | no other type | 62/222 (27.9%) | 53/221 (24.0%) | 63/238 (26.5%) | 61/237 (25.7%) |
| **Sanitation** | Any latrine | 73/222 (32.9%) | 88/220 (40.0%) | 96/234 (41.0%) | 88/227 (38.8%) |
|  | Improved latrine | 60/222 (27.0%) | 73/220 (33.2%) | 83/233 (35.6%) | 75/227 (33.0%) |
| **Water** | Main source of household drinking water is improved | 148/222 (66.7%) | 155/220 (70.5%) | 154/233 (66.1%) | 163/229 (71.2%) |
|  | Treat drinking water to make it safer | 39/221 (17.7%) | 35/219 (16.0%) | 36/232 (15.5%) | 19/227 (33.0%) |
|  | 1 way walk time to fetch water | 10.0 (5.0 ; 20.0) | 10.0 (5.0 ; 20.0) | 10.0 (5.0 ; 20.0) | 10.0 (5.0 ; 20.0) |
|  | Per capita water volume | 6.7 (4.2 ; 10.0) | 6.7 (4.0 ; 10.0) | 6.7 (5.0 ; 10.0) | 6.7 (4.4 ; 10.0) |
| **Hygiene** | Handwashing station with water | 16/216 (7.4%) | 11/214 (5.1%) | 36/229 (15.7%) | 38/221 (17.2%) |
|  | Improved floor | 116/217 (53.5%) | 115/220 (52.3%) | 137/233 (58.8%) | 119/234 (8.4%) |
|  | Chickens yes/no | 177/224 (79.0%) | 187/222 (84.2%) | 190/236 (80.5%) | 199/237 (84.0%) |
|  | Faeces observed in yard | 75/239 (31.4%) | 92/242 (38.0%) | 87/240 (36.3%) | 69/241 (28.6%) |
| **Diet quality and food security** | Household meets minimum dietary diversity score | 76/213 (35.7%) | 87/214 (40.7%) | 83/227 (36.6%) | 85/230 (37.0%) |
|  | Coping Strategies index | 1.0 (0.0 ; 6.0) | 0.0 (0.0 ; 5.0) | 0.0 (0.0 ; 5.0) | 1.0 (0.0 ; 6.0) |
| **Maternal characteristics** | Age ( yr, SD)d | 25.3 (6.3) | 25.5 (6.0) | 25.8 96.8) | 26.2 (5.9) |
|  | Maternal height | 159.9 (5.9) | 160.1 (6.4) | 159.6 (5.6%) | 159.9 (5.9) |
|  | Maternal MUAC | 26.2 (3.1) | 26.6 (3.2) | 26.8 (3.6) | 26.7 (3.1) |
|  | Maternal Schooling | 9.7 (1.8) | 9.9 (1.6) | 9.6 (1.6) | 9.6 (1.7) |
|  | Parity | 2.0 (1.0 ; 3.0) | 1.0 (1.0 ; 2.0) | 1.0 (1.0 ; 3.0) | 2.0 (1.0 ; 3.0) |
|  | Married | 226/240 (51.0%) | 219/232 (94.4%) | 220/231 (95.2%) | 228/236 (96.6%) |
|  | Employed | 10/223 (4.5%) | 20/222 (9.0%) | 24/238 (10.1%) | 16/237 (6.8%) |
|  | Religion |  |  |  |  |
|  | Apostolic | 123/241 (51.0%) | 111/233 (47.6%) | 112/234 (47.9%) | 110/237 (46.4%) |
|  | Other Christian | 101/241 (41.9%) | 107/233 (45.9%) | 90/234 (38.5%) | 100/237 (42.2%) |
|  | Other religion | 17/241 (7.1%) | 15/233 (6.4%) | 32/234 (13.7%) | 27/237 (11.4%) |
| **Maternal capabilities** | Gender norm attitudes | 2.7 (1.7 ; 3.2) | 2.7 (1.7 ; 3.2) | 1.7 (1.5 ; 3.0) | 2.0 (1.5 ; 3.0) |
|  | Perceived social support | 3.5 (3.1 ; 3.9) | 3.7 (3.1 ; 4.1) | 3.6 (3.2 ; 3.1) | 3.7 (3.2 ; 4.0) |
| **Child characteristics** | Female | 120/251 (47.8%) | 121/251 (48.2%) | 141/250 (56.4%) | 129/250 (51.6%) |
|  | Birthweight | 3.1 (0.5) | 3.1 (0.5) | 3.1 (0.5) | 3.1 (0.4) |
|  | Low birthweight | 25/237 (10.6%) | 20/240 (8.3%) | 28/242 (11.6%) | 17/238 (7.1%) |
|  | Institutional delivery | 213/238 (89.5%) | 215/232 (92.7%) | 211/235 (89.8%) | 216/230 (93.9%) |
|  | Vaginal delivery | 235/246 (95.5%) | 224/236 994.9%) | 231/245 (94.3%) | 219/238 (92.0%) |
|  | LAZ at 18 months | -1.6 (1.0) | -1.4 (1.1) | -1.6 (1.0) | -1.5 (1.0) |
|  | Stunted at 18 months | 87/249 (34.9%) | 61/249 (24.5%) | 80/247 32.4%) | 70/248 (28.2%) |
|  | WAZ at 18 months | -0.8 (1.0) | -0.6 (1.0) | -0.8 (1.0) | -0.8 (0.9) |
|  | headcirc at 18 months | -0.2 (1.0) | -0.2 (1.0) | -03 (1.1) | -0.2 (1.1) |
|  | MUAC at 18 months | 0.2 (0.9) | 0.2 (0.9) | -0.01 (0.9) | 0.1 (0.8) |
|  | MDAT total at 24 months, n, mean, SD | 91.1 (11.0) | 93.1 (9.2) | 90/7 (9.9) | 92.7 (10.0) |
|  | Mcarthur Bates at 24 months, n, mean, SD | 60.5 (21.0) | 64.1 (18.1) | 61.1 (20.8) | 63.0 (20.4) |

Table S2: Baseline characteristics of SHINE Follow-up households for children born from HIV negative mothers (CHU) split by SHINE intervention arm. Household, maternal and infant characteristics are included.

### Table S3: effect of intervention by child sex

| **Variable** |  | **P-value of interaction of intervention with child sex** | **GEE Coefficient for Girls (95% CI)** | p-value | **GEE coefficient for Boys (95% CI)** | p-value |
| --- | --- | --- | --- | --- | --- | --- |
| Mental Processing Index | IYCF | 0.062 | N/A |  | N/A |  |
|  | WASH | 0.280 | N/A |  | N/A |  |
| School Achievement Test | IYCF | 0.528 | N/A |  | N/A |  |
|  | WASH | 0.773 | N/A |  | N/A |  |
| Plus EF test score | IYCF | 0.091 | N/A |  | N/A |  |
|  | WASH | 0.132 | N/A |  | N/A |  |
| Fine motor , sec | IYCF | 0.227 | N/A |  | N/A |  |
|  | WASH | 0.355 | N/A |  | N/A |  |
| Strengths and Difficulties Questionnaire | IYCF | 0.628 | N/A |  | N/A |  |
|  | WASH | 0.905 | N/A |  | N/A |  |
| Child socioemotional score | IYCF | 0.795 | N/A |  | N/A |  |
|  | WASH | 0.950 | N/A |  | N/A |  |
| Mean Grip Strength, Kg | IYCF | 0.025 | -0.23 (-0.62, 0.16) | 0.251 | 0.53 (0.19, 0.87) | 0.002 |
|  | WASH | 0.292 | N/A |  | N/A |  |
|  | IYCF&WASH | 0.102 | N/A |  | N/A |  |
| Mean Broad jump, m | IYCF | 0.553 | N/A |  | N/A |  |
|  | WASH | 0.760 | N/A |  | N/A |  |
|  | IYCF&WASH | 0.895 | N/A |  | N/A |  |
| VO2max (Cardiovascular fitness) | IYCF | 0.079 | N/A |  | N/A |  |
|  | WASH | 0.126 | N/A |  | N/A |  |
|  | IYCF&WASH | 0.038 | 0.58 (0.07, 1.08) | 0.025 | -0.29 (-0.87, 0.29) | 0.324 |
| Diastolic BP, mm Hg | IYCF | 0.917 | N/A |  | N/A |  |
|  | WASH | 0.965 | N/A |  | N/A |  |
|  | IYCF&WASH | 0.999 | N/A |  | N/A |  |
| Systolic BP, mm Hg | IYCF | 0.485 | N/A |  | N/A |  |
|  | WASH | 0.997 | N/A |  | N/A |  |
|  | IYCF&WASH | 0.590 | N/A |  | N/A |  |
| Height-for-age Z-score | IYCF | 0.251 | N/A |  | N/A |  |
|  | WASH | 0.836 | N/A |  | N/A |  |
| Weight-for-age Z-score | IYCF | 0.961 | N/A |  | N/A |  |
|  | WASH | 0.694 | N/A |  | N/A |  |
| BMI Z-score | IYCF | 0.210 | N/A |  | N/A |  |
|  | WASH | 0.831 | N/A |  | N/A |  |
| Knee-heel length | IYCF | 0.111 | N/A |  | N/A |  |
|  | WASH | 0.944 | N/A |  | N/A |  |
| Head circ, cm | IYCF | 0.402 | N/A |  | N/A |  |
|  | WASH | 0.634 | N/A |  | N/A |  |
| MUAC, cm | IYCF | 0.457 | N/A |  | N/A |  |
|  | WASH | 0.478 | N/A |  | N/A |  |
| Waist circ, cm | IYCF | 0.641 | N/A |  | N/A |  |
|  | WASH | 0.779 | N/A |  | N/A |  |
| Hip circ, cm | IYCF | 0.254 | N/A |  | N/A |  |
|  | WASH | 0.941 | N/A |  | N/A |  |
| Calf circ, m | IYCF | 0.524 | N/A |  | N/A |  |
|  | WASH | 0.799 | N/A |  | N/A |  |
| Lean mass index, Ohms^-1^ | IYCF | 0.349 | N/A |  | N/A |  |
|  | WASH | 0.231 | N/A |  | N/A |  |
| Impedance Index, m^2^ Ohms^-1^ | IYCF | 0.993 | N/A |  | N/A |  |
|  | WASH | 0.531 | N/A |  | N/A |  |
| Phase angle, degrees | IYCF | 0.566 | N/A |  | N/A |  |
|  | WASH | 0.739 | N/A |  | N/A |  |
| Total skinfold thicknesses, mm | IYCF | 0.585 | N/A |  | N/A |  |
|  | WASH | 0.999 | N/A |  | N/A |  |
| Peripheral skinfold thickness, mm | IYCF | 0.385 | N/A |  | N/A |  |
|  | WASH | 0.823 | N/A |  | N/A |  |
| Central skinfold thickness, mm | IYCF | 0.986 | N/A |  | N/A |  |
|  | WASH | 0.924 | N/A |  | N/A |  |
| Hb, g dl^-1^ | IYCF | 0.976 | N/A |  | N/A |  |
|  | WASH | 0.121 | N/A |  | N/A |  |

Table S3: Pre-specified sub-analysis on the effect of intervention by child sex.

###

### Table S4-1 to S4-5: Long-term effects of randomised interventions on Cognition subtests

|  |  | Effects by arm | | |  | Main effects | | | | | | | |
| --- | --- | --- | --- | --- | --- | --- | --- | --- | --- | --- | --- | --- | --- |
| Test | Detailed Outcome | Treatment group | N | Mean (SD) | Treatment Group | | N | Mean (SD) | Unadjusted diff (95% CI) | p | N Adj | Adjusted diff (95%CI) | p |
| Kaufmann Assessment Battery for Children 2^nd^ edition (KABC-II) subtests | Atlantis | SoC | 246 | 6 (2) | No IYCF | | 493 | 6 (2) | 0.0 (ref) |  |  | 0.0 (ref) |  |
|  |  | IYCF | 250 | 6 (2) | IYCF | | 497 | 6 (3) | 0 (-1, 0) | 0.103 | 980 | 0 (-1, 0) | 0.073 |
|  |  | WASH | 247 | 6 (2) | No WASH | | 496 | 6 (2) | 0.0 (ref) |  |  | 0.0 (ref) |  |
|  |  | WASH & IYCF | 247 | 6 (3) | WASH | | 494 | 6 (2) | 0 (0, 0) | 0.503 | 980 | 0 (0, 0) | 0.697 |
|  | Story Completion | SoC | 246 | 5 (2) | No IYCF | | 493 | 5 (2) | 0.0 (ref) |  |  | 0.0 (ref) |  |
|  |  | IYCF | 250 | 5 (2) | IYCF | | 497 | 5 (2) | 0 (0, 0) | 0.964 | 980 | 0 (0, 0) | 0.608 |
|  |  | WASH | 247 | 5 (2) | No WASH | | 496 | 5 (2) | 0.0 (ref) |  |  | 0.0 (ref) |  |
|  |  | WASH & IYCF | 247 | 4 (2) | WASH | | 494 | 4 (2) | 0 (0, 0) | 0.653 | 980 | 0 (0, 0) | 0.684 |
|  | Number recall | SoC | 246 | 7 (2) | No IYCF | | 493 | 7 (2) | 0.0 (ref) |  |  | 0.0 (ref) |  |
|  |  | IYCF | 250 | 8 (2) | IYCF | | 497 | 7 (2) | 0 (0, 0) | 0.736 | 980 | 0 (0, 0) | 0.718 |
|  |  | WASH | 247 | 7 (2) | No WASH | | 496 | 8 (2) | 0.0 (ref) |  |  | 0.0 (ref) |  |
|  |  | WASH & IYCF | 247 | 7 (2) | WASH | | 494 | 7 (2) | 0 (0, 0) | 0.100 | 980 | 0 (0, 0) | 0.206 |
|  | Atlantis Delayed | SoC | 246 | 7 (2) | No IYCF | | 493 | 7 (2) | 0.0 (ref) |  |  | 0.0 (ref) |  |
|  |  | IYCF | 250 | 7 (2) | IYCF | | **497** | **7 (2)** | **0 (-1, 0)** | **0.010** | **980** | **0 (-1, 0)** | **0.011** |
|  |  | WASH | 247 | 7 (2) | No WASH | | 496 | 7 (2) | 0.0 (ref) |  |  | 0.0 (ref) |  |
|  |  | WASH & IYCF | 247 | 7 (2) | WASH | | 494 | 7 (2) | 0 (0, 0) | 0.791 | 980 | 0 (0, 0) | 0.981 |
|  | Rover | SoC | 246 | 7 (2) | No IYCF | | 493 | 7 (2) | 0.0 (ref) |  |  | 0.0 (ref) |  |
|  |  | IYCF | 250 | 7 (2) | IYCF | | 497 | 7 (2) | 0 (-1, 0) | 0.382 | 980 | 0 (-1, 0) | 0.264 |
|  |  | WASH | 247 | 7 (2) | No WASH | | 496 | 7 (2) | 0.0 (ref) |  |  | 0.0 (ref) |  |
|  |  | WASH & IYCF | 247 | 7 (2) | WASH | | 494 | 7 (2) | 0 (0, 0) | 0.891 | 980 | 0 (0, 0) | 0.805 |
|  | Triangle | SoC | 246 | 4 (2) | No IYCF | | 493 | 4 (2) | 0.0 (ref) |  |  | 0.0 (ref) |  |
|  |  | IYCF | 250 | 4 (2) | IYCF | | 497 | 4 (2) | 0 (0, 0) | 0.381 | 980 | 0 (0, 0) | 0.415 |
|  |  | WASH | 247 | 4 (2) | No WASH | | 496 | 4 (2) | 0.0 (ref) |  |  | 0.0 (ref) |  |
|  |  | WASH & IYCF | 247 | 4 (2) | WASH | | 494 | 4 (2) | 0 (0, 0) | 0.633 | 980 | 0 (0, 0) | 0.385 |
|  | Word Order | SoC | 246 | 6 (2) | No IYCF | | 493 | 6 (2) | 0.0 (ref) |  |  | 0.0 (ref) |  |
|  |  | IYCF | 250 | 6 (2) | IYCF | | 497 | 6 (2) | 0 (0, 0) | 0.071 | 980 | 0 (0, 0) | 0.063 |
|  |  | WASH | 247 | 6 (2) | No WASH | | 496 | 6 (2) | 0.0 (ref) |  |  | 0.0 (ref) |  |
|  |  | WASH & IYCF | 247 | 6 (2) | WASH | | 494 | 6 (2) | 0 (0, 0) | 0.613 | 980 | 0 (0, 0) | 0.497 |
|  | Pattern Reasoning | SoC | 246 | 6 (3) | No IYCF | | 493 | 6 (3) | 0.0 (ref) |  |  | 0.0 (ref) |  |
|  |  | IYCF | 250 | 6 (2) | IYCF | | 497 | 6 (3) | 0 (0, 0) | 0.858 | 980 | 0 (0, 0) | 0.954 |
|  |  | WASH | 247 | 6 (2) | No WASH | | 496 | 6 (3) | 0.0 (ref) |  |  | 0.0 (ref) |  |
|  |  | WASH & IYCF | 247 | 6 (3) | WASH | | 494 | 6 (3) | 0 (-1, 0) | 0.282 | 980 | 0 (-1, 0) | 0.382 |
| KABC-II cognitive sub-domains | Learn | SoC | 246 | 14 (4) | No IYCF | | 493 | 14 (4) | 0.0 (ref) |  |  | 0.0 (ref) |  |
|  |  | IYCF | 250 | 13 (4) | IYCF | | 497 | 13 (4) | **-1 (-1, 0)** | **0.030** | **980** | **-1 (-1, 0)** | **0.022** |
|  |  | WASH | 247 | 14 (4) | No WASH | | 496 | 13 (4) | 0.0 (ref) |  |  | 0.0 (ref) |  |
|  |  | WASH & IYCF | 247 | 13 (4) | WASH | | 494 | 13 (4) | 0 (-1, 0) | 0.494 | 980 | 0 (-1, 0) | 0.721 |
|  | Planning | SoC | 246 | 11 (4) | No IYCF | | 493 | 11 (4) | 0.0 (ref) |  |  | 0.0 (ref) |  |
|  |  | IYCF | 250 | 11 (3) | IYCF | | 497 | 11 (3) | 0 (0, 0) | 0.995 | 980 | 0 (-1, 0) | 0.713 |
|  |  | WASH | 247 | 10 (4) | No WASH | | 496 | 11 (4) | 0.0 (ref) |  |  | 0.0 (ref) |  |
|  |  | WASH & IYCF | 247 | 10 (4) | WASH | | 494 | 10 (4) | 0 (-1, 0) | 0.243 | 980 | 0 (-1, 0) | 0.376 |
|  | Simultaneous | SoC | 246 | 11 (4) | No IYCF | | 493 | 11 (4) | 0.0 (ref) |  |  | 0.0 (ref) |  |
|  |  | IYCF | 250 | 11 (4) | IYCF | | 497 | 11 (3) | 0 (-1, 1) | 0.986 | 980 | 0 (-1, 1) | 0.874 |
|  |  | WASH | 247 | 11 (4) | No WASH | | 496 | 11 (4) | 0.0 (ref) |  |  | 0.0 (ref) |  |
|  |  | WASH & IYCF | 247 | 11 (3) | WASH | | 494 | 11 (4) | 0 (-1, 0) | 0.700 | 980 | 0 (-1, 0) | 0.512 |
|  | Sequential | SoC | 246 | 13 (4) | No IYCF | | 485 | 3.7 (0.7) | 0.0 (ref) |  |  | 0.0 (ref) |  |
|  |  | IYCF | 250 | 13 (4) | IYCF | | 488 | 3.7 (0.7) | 0 (-1, 0) | 0.328 | 980 | 0 (-1, 0) | 0.265 |
|  |  | WASH | 247 | 13 (4) | No WASH | | 489 | 3.7 (0.7) | 0.0 (ref) |  |  | 0.0 (ref) |  |
|  |  | WASH & IYCF | 247 | 13 (4) | WASH | | 484 | 3.7 (0.7) | 0 (-1, 0) | 0.210 | 980 | 0 (-1, 0) | 0.205 |

Table S4-1 Kaufman Assessment Battery for Children 2^nd^ edition (KABC-II) individual subtest and domain scores

| Test | Detailed Outcome | Treatment group | N | Mean (SD) | Treatment Group | N | Mean (SD) | Unadjusted diff (95% CI) | p | N Adj | Adjusted diff (95%CI) | p |
| --- | --- | --- | --- | --- | --- | --- | --- | --- | --- | --- | --- | --- |
| School Achievement Test Scores | Numeracy | SoC | 246 | 19 (6) | No IYCF | 493 | 19 (6) | 0.0 (ref) |  | 0.0 (ref) |  |  |
|  |  | IYCF | 250 | 18 (6) | IYCF | 497 | 18 (6) | 0 (-1, 1) | 0.433 | 0 (-1, 1) | 0.462 | 980 |
|  |  | WASH | 247 | 18 (6) | No WASH | 496 | 19 (6) | 0.0 (ref) |  | 0.0 (ref) |  |  |
|  |  | WASH & IYCF | 247 | 18 (6) | WASH | 494 | 18 (6) | -1 (-2, 0) | 0.162 | -1 (-2, 0) | 0.163 | 980 |
|  | Reading | SoC | 246 | 14 (14) | No IYCF | 493 | 14 (14) | 0.0 (ref) |  | 0.0 (ref) |  |  |
|  |  | IYCF | 250 | 13 (13) | IYCF | 497 | 13 (13) | -1 (-3, 1) | 0.332 | -1 (-3, 1) | 0.376 | 980 |
|  |  | WASH | 247 | 14 (14) | No WASH | 496 | 14 (13) | 0.0 (ref) |  | 0.0 (ref) |  |  |
|  |  | WASH & IYCF | 247 | 12 (13) | WASH | 494 | 13 (13) | -1 (-3, 1) | 0.476 | -1 (-3, 2) | 0.585 | 980 |
|  | Writing | SoC | 246 | 14 (10) | No IYCF | 493 | 14 (10) | 0.0 (ref) |  | 0.0 (ref) |  |  |
|  |  | IYCF | 250 | 14 (10) | IYCF | 497 | 14 (10) | 0 (-2, 1) | 0.738 | 0 (-2, 1) | 0.822 | 980 |
|  |  | WASH | 247 | 14 (10) | No WASH | 496 | 14 (10) | 0.0 (ref) |  | 0.0 (ref) |  |  |
|  |  | WASH & IYCF | 247 | 13 (9) | WASH | 494 | 14 (10) | -1 (-2, 1) | 0.332 | -1 (-2, 1) | 0.364 | 980 |

Table S4-2 School Achievement (SAT) subtest scores

| Test | Detailed Outcome | Treatment group | N | Mean (SD) | Treatment Group | N | Mean (SD) | Unadjusted diff (95% CI) | p | N Adj | Adjusted diff (95%CI) | p |
| --- | --- | --- | --- | --- | --- | --- | --- | --- | --- | --- | --- | --- |
| Plus EF subtest scores | MSIT | SoC | 240 | 25 (10) | No IYCF | 486 | 24 (11) | 0.0 (ref) |  | 0.0 (ref) |  |  |
|  |  | IYCF | 248 | 25 (11) | IYCF | 492 | 25 (11) | 1 (-1, 2) | 0.343 | 1 (-1, 3) | 0.218 | 968 |
|  |  | WASH | 246 | 24 (11) | No WASH | 488 | 25 (10) | 0.0 (ref) |  | 0.0 (ref) |  |  |
|  |  | WASH & IYCF | 244 | 24 (11) | WASH | 490 | 24 (11) | -1 (-3, 0) | 0.124 | -1 (-3, 0) | 0.136 | 968 |
|  | Stars and Flowers | SoC | 240 | 43 (9) | No IYCF | 486 | 43 (9) | 0.0 (ref) |  | 0.0 (ref) |  |  |
|  |  | IYCF | 248 | 43 (9) | IYCF | 492 | 43 (9) | 0 (-1, 1) | 0.970 | 0 (-1, 1) | 0.929 | 968 |
|  |  | WASH | 246 | 43 (9) | No WASH | 488 | 43 (9) | 0.0 (ref) |  | 0.0 (ref) |  |  |
|  |  | WASH & IYCF | 244 | 42 (9) | WASH | 490 | 42 (9) | -1 (-2, 0) | 0.214 | -1 (-2, 0) | 0.151 | 968 |
|  | Flanker | SoC | 240 | 47 (12) | No IYCF | 486 | 47 (12) | 0.0 (ref) |  | 0.0 (ref) |  |  |
|  |  | IYCF | 248 | 47 (11) | IYCF | 492 | 48 (11) | 1 (0, 2) | 0.185 | 1 (0, 2) | 0.193 | 968 |
|  |  | WASH | 246 | 46 (12) | No WASH | 488 | 47 (12) | 0.0 (ref) |  | 0.0 (ref) |  |  |
|  |  | WASH & IYCF | 244 | 48 (11) | WASH | 490 | 47 (12) | 0 (-2, 1) | 0.752 | 0 (-2, 1) | 0.661 | 968 |
|  |  |  |  |  |  |  |  |  |  |  |  |  |

Table S4-3 Plus EF subtest scores

| Test | Detailed Outcome | Treatment group | N | Mean (SD) | Treatment Group | N | Mean (SD) | Unadjusted diff (95% CI) | p | N Adj | Adjusted diff (95%CI) | p |
| --- | --- | --- | --- | --- | --- | --- | --- | --- | --- | --- | --- | --- |
| Finger tapping time, seconds | Dominant Hand | SoC | 244 | 23.1 (6.5) | No IYCF | 491 | 23 (6.3) | 0.0 (ref) |  | 0.0 (ref) |  |  |
|  |  | IYCF | 250 | 23.8 (7.3) | IYCF | 495 | 23.7 (7.3) | 0.7 (-0.3, 1.6) | 0.168 | 0.6 (-0.3, 1.5) | 0.169 | 976 |
|  |  | WASH | 247 | 22.8 (6) | No WASH | 494 | 23.5 (6.9) | 0.0 (ref) |  | 0.0 (ref) |  |  |
|  |  | WASH & IYCF | 245 | 23.6 (7.2) | WASH | 492 | 23.2 (6.6) | -0.3 (-1.2, 0.6) | 0.544 | -0.6 (-1.5, 0.4) | 0.22 | 976 |
|  | Non- dominant hand | SoC | 244 | 24.4 (7.0) | No IYCF | 491 | 24.4 (6.8) | 0.0 (ref) |  | 0.0 (ref) |  |  |
|  |  | IYCF | 250 | 25.6 (7.6) | IYCF | 495 | 25.2 (7.3) | 0.8 (-0.1, 1.7) | 0.069 | 0.8 (-0.1, 1.7) | 0.066 | 976 |
|  |  | WASH | 247 | 24.3 (6.5) | No WASH | 494 | 25.0 (7.3) | 0.0 (ref) |  | 0.0 (ref) |  |  |
|  |  | WASH & IYCF | 245 | 24.8 (7.1) | WASH | 492 | 24.6 (6.8) | -0.4 (-1.3, 0.5) | 0.346 | -0.7 (-1.6, 0.2) | 0.151 | 976 |

Table S4-4 Fine motor finger tapping subtest scores

| Test | Detailed Outcome | Treatment group | N | Mean (SD) | Treatment Group | N | Mean (SD) | Unadjusted diff (95% CI) | p | N Adj | Adjusted diff (95%CI) | p |
| --- | --- | --- | --- | --- | --- | --- | --- | --- | --- | --- | --- | --- |
| Strengths and Difficulties Questionnaire sub-scales | Emotional Problems Scale | SoC | 245 | 2 (2) | No IYCF | 492 | 2 (2) | 0.0 (ref) |  |  |  |  |
|  |  | IYCF | 250 | 2 (2) | IYCF | 497 | 2 (2) | 0 (-1, 0) | 0.168 | 0 (-1, 0) | 0.223 | 979 |
|  |  | WASH | 247 | 2 (2) | No WASH | 495 | 2 (2) |  |  |  |  |  |
|  |  | WASH & IYCF | 247 | 2 (2) | WASH | 494 | 2 (2) | 0 (-1, 0) | 0.014 | 0 (-1, 0) | 0.031 | 979 |
|  | Conduct Problems Scale | SoC | 245 | 2 (2) | No IYCF | 492 | 2 (2) | 0.0 (ref) |  |  |  |  |
|  |  | IYCF | 250 | 2 (2) | IYCF | 497 | 2 (2) | 0 (0, 0) | 0.163 | 0 (0, 0) | 0.151 | 979 |
|  |  | WASH | 247 | 2 (2) | No WASH | 495 | 2 (2) |  |  |  |  |  |
|  |  | WASH & IYCF | 247 | 2 (2) | WASH | 494 | 2 (2) | 0 (0, 0) | 0.115 | 0 (-1, 0) | 0.088 | 979 |
|  | Hyperactivity Scale | SoC | 245 | 4 (2) | No IYCF | 492 | 4 (2) | 0.0 (ref) |  |  |  |  |
|  |  | IYCF | 250 | 4 (2) | IYCF | 497 | 4 (2) | 0 (0, 0) | 0.323 | 0 (-1, 0) | 0.248 | 979 |
|  |  | WASH | 247 | 4 (2) | No WASH | 495 | 4 (2) |  |  |  |  |  |
|  |  | WASH & IYCF | 247 | 3 (2) | WASH | 494 | 3 (2) | 0 (-1, 0) | 0.053 | 0 (-1, 0) | 0.089 | 979 |
|  | Peer Problems Scale | SoC | 245 | 1 (2) | No IYCF | 492 | 1 (1) | 0.0 (ref) |  |  |  |  |
|  |  | IYCF | 250 | 1 (1) | IYCF | 497 | 1 (1) | 0 (0, 0) | 0.157 | 0 (0, 0) | 0.387 | 979 |
|  |  | WASH | 247 | 1 (1) | No WASH | 495 | 1 (1) |  |  |  |  |  |
|  |  | WASH & IYCF | 247 | 1 (1) | WASH | 494 | 1 (1) | 0 (0, 0) | 0.648 | 0 (0, 0) | 0.27 | 979 |
|  | Prosocial Scale | SoC | 245 | 8 (2) | No IYCF | 492 | 8 (2) |  |  |  |  |  |
|  |  | IYCF | 250 | 8 (2) | IYCF | 497 | 8 (2) | 0 (0, 0) | 0.239 | 0 (0, 0) | 0.272 | 979 |
|  |  | WASH | 247 | 8 (2) | No WASH | 495 | 8 (2) |  |  |  |  |  |
|  |  | WASH & IYCF | 247 | 8 (2) | WASH | 494 | 8 (2) | 0 (0, 0) | 0.161 | 0 (0, 0) | 0.49 | 979 |

Table S4-5 Strength and Difficulties subtest scores

| Test | Detailed Outcome | Treatment group | N | Mean (SD) | Treatment Group | N | Mean (SD) | Unadjusted diff (95% CI) | p | N Adj | Adjusted diff (95%CI) | p |
| --- | --- | --- | --- | --- | --- | --- | --- | --- | --- | --- | --- | --- |
| Child Socioemtional Questionnaire | Child Socioemotional Questionnaire without food security question | SoC | 242 | 4 (1) | No IYCF | 485 | 4 (1) |  |  |  |  |  |
|  |  | IYCF | 247 | 4 (1) | IYCF | 488 | 4 (1) | 0 (0,0) | 0.705 | 0 (0, 0) | 0.84 | 963 |
|  |  | WASH | 243 | 4 (1) | No WASH | 489 | 4 (1) |  |  |  |  |  |
|  |  | WASH & IYCF | 241 | 4 (1) | WASH | 484 | 4 (1) | 0 (0,0) | 0.255 | 0 (0, 0) | 0.251 | 963 |

Table S4-6 Child’s own socioemotional subtest score without food security question

### Table S5-1 to S5-2: Secondary physical function outcomes

| Outcome | Treatment group | Number | Mean (SD) | Unadjusted diff (95%CI) | p | Adjusted diff (95%CI) | p | N Adj |
| --- | --- | --- | --- | --- | --- | --- | --- | --- |
| Grip strength Dominant Hand, Kg | SoC | 246 | 10.6 (1.9) | 0.0 (ref) |  |  |  |  |
|  | IYCF | 250 | 10.9 (2.1) | 0.3 (0.1, 0.5) | 0.008 | 0.3 (0.1, 0.5) | 0.007 | 980 |
|  | WASH | 247 | 10.9 (2.1) | 0.2 (-0.2, 0.5) | 0.303 | 0.1 (-0.2, 0.4) | 0.532 | 980 |
|  | WASH & IYCF | 247 | 10.8 (2) | 0.1 (-0.2, 0.4) | 0.398 | 0.2 (0, 0.4) | 0.117 | 980 |
| Grip Strength Non-dominant Hand, Kg | SoC | 246 | 10.5 (2) | 0.0 (ref) |  |  |  |  |
|  | IYCF | 250 | 10.7 (2.2) | 0.2 (-0.1, 0.5) | 0.115 | 0.2 (-0.1, 0.5) | 0.212 | 980 |
|  | WASH | 247 | 10.6 (2.3) | 0.1 (-0.3, 0.4) | 0.725 | 0 (-0.3, 0.4) | 0.862 | 980 |
|  | WASH & IYCF | 247 | 10.5 (2) | 0 (-0.3, 0.3) | 0.893 | 0 (-0.3, 0.3) | 0.895 | 980 |
| Standardised Grip strength (a) | SoC | 246 | 0 (0.9) | 0.0 (ref) |  |  |  |  |
|  | IYCF | 250 | 0.1 (1) | 0.1 (0, 0.3) | 0.032 | 0.1 (0, 0.2) | 0.056 | 980 |
|  | WASH | 247 | 0.1 (1) | 0.1 (-0.1, 0.2) | 0.421 | 0 (-0.1, 0.2) | 0.8 | 980 |
|  | WASH & IYCF | 247 | 0 (1) | 0 (-0.1, 0.2) | 0.734 | 0 (-0.1, 0.1) | 0.616 | 980 |
| Standardised Broad jump (b) | SoC | 245 | 0 (0.9) | 0.0 (ref) |  |  |  |  |
|  | IYCF | 249 | 0 (1) | 0 (-0.2, 0.1) | 0.683 | -0.1 (-0.2, 0.1) | 0.517 | 977 |
|  | WASH | 246 | 0.1 (0.9) | 0 (-0.2, 0.2) | 0.922 | 0 (-0.2, 0.2) | 0.993 | 977 |
|  | WASH & IYCF | 247 | 0.1 (1) | 0 (-0.2, 0.2) | 0.899 | 0 (-0.2, 0.2) | 0.875 | 977 |
| Standardised VO2max (c) | SoC | 245 | 0.2 (1) | 0.0 (ref) |  |  |  |  |
|  | IYCF | 248 | 0 (0.9) | -0.1 (-0.3, 0.1) | 0.183 | -0.2 (-0.3, 0) | 0.104 | 975 |
|  | WASH | 247 | 0 (1.1) | -0.2 (-0.4, 0) | 0.101 | -0.1 (-0.3, 0.1) | 0.38 | 975 |
|  | WASH & IYCF | 246 | 0.1 (1) | -0.1 (-0.2, 0.1) | 0.558 | 0 (-0.2, 0.2) | 0.758 | 975 |
| Standardised physical function score (= a + b +c) | SoC | 244 | 0.2 (2) | 0.0 (ref) |  |  |  |  |
|  | IYCF | 248 | 0.1 (2.1) | -0.1 (-0.4, 0.3) | 0.746 | -0.1 (-0.5, 0.2) | 0.523 | 978 |
|  | WASH | 246 | 0.1 (2.1) | -0.1 (-0.5, 0.4) | 0.708 | 0 (-0.4, 0.5) | 0.863 | 978 |
|  | WASH & IYCF | 246 | 0.1 (2) | 0.0 (-0.4, 0.4) | 0.913 | 0 (-0.4, 0.4) | 0.98 | 978 |

Table S5-1: Secondary strength and fitness outcomes

| Resting pulse pressure, mm Hg | SoC | 245 | 34.8 (7.8) | 0.0 (ref) |  |  |  |  |
| --- | --- | --- | --- | --- | --- | --- | --- | --- |
|  | IYCF | 250 | 34.4 (7.4) | -0.5 (-1.6, 0.6) | 0.396 | -0.3 (-1.1, 0.6) | 0.558 | 978 |
|  | WASH | 246 | 34.7 (7.5) | 0 (-1, 1) | 0.968 | 0 (-0.9, 0.9) | 0.998 | 978 |
|  | WASH & IYCF | 247 | 34.8 (7.2) | -0.1 (-1.2, 1.1) | 0.927 | -0.2 (-1.5, 1.1) | 0.751 | 978 |
| Systolic blood pressure 1 minute after shuttle run test, mm Hg | SoC | 242 | 127.1 (10) | 0.0 (ref) |  |  |  |  |
|  | IYCF | 247 | 126.9 (10.2) | -0.2 (-2.1, 1.7) | 0.83 | 0 (-1.9, 1.9) | 0.981 | 966 |
|  | WASH | 245 | 125.2 (11.6) | -1.9 (-3.7, -0.2) | 0.028 | -1.5 (-3.1, 0.1) | 0.072 | 966 |
|  | WASH & IYCF | 242 | 126.9 (10.4) | -0.3 (-2.1, 1.6) | 0.786 | -0.3 (-2.1, 1.5) | 0.764 | 966 |
| Diastolic Blood pressure 1 minuted after shuttle run test, mm Hg | SoC | 245 | 85.9 (10.3) | 0.0 (ref) |  |  |  |  |
|  | IYCF | 248 | 85.5 (11.3) | -0.5 (-2.1, 1.2) | 0.6 | -0.5 (-2.2, 1.2) | 0.574 | 975 |
|  | WASH | 246 | 85.9 (12.2) | 0.1 (-1.6, 1.8) | 0.932 | 0.3 (-1.4, 2) | 0.727 | 975 |
|  | WASH & IYCF | 246 | 85.8 (11.9) | 0.1 (-1.8, 2) | 0.927 | 0 (-1.8, 1.9) | 0.972 | 975 |
| Exercise pulse pressure, mm Hg | SoC | 245 | 40.2 (8.6) | 0.0 (ref) |  |  |  |  |
|  | IYCF | 248 | 40.4 (8.6) | 0.1 (-1, 1.3) | 0.796 | 0.2 (-0.9, 1.3) | 0.763 | 975 |
|  | WASH | 246 | 39.3 (8.8) | -1 (-1.9, 0) | 0.052 | -0.4 (-1.5, 0.7) | 0.47 | 975 |
|  | WASH & IYCF | 246 | 40 (8.5) | -0.2 (-1.4, 1) | 0.739 | -0.3 (-1.3, 0.6) | 0.474 | 975 |
| Change in systolic blood pressure between 1st and 5th readings after Shuttle run test, mm Hg | SoC | 242 | 22.1 (7.8) | 0.0 (ref) |  |  |  |  |
|  | IYCF | 247 | 22.3 (8.4) | 0.1 (-1.4, 1.7) | 0.857 | 0.1 (-1.3, 1.5) | 0.924 | 965 |
|  | WASH | 244 | 21.7 (8.2) | -0.4 (-1.7, 0.9) | 0.562 | -0.3 (-1.4, 0.8) | 0.646 | 965 |
|  | WASH & IYCF | 242 | 21.4 (8.4) | -0.7 (-2.1, 0.6) | 0.288 | -0.6 (-1.5, 0.4) | 0.235 | 965 |
| Change in diastolic blood pressure between 1st and 5th readings after Shuttle run test, mm Hg | SoC | 245 | 18 (7) | 0.0 (ref) |  |  |  |  |
|  | IYCF | 248 | 18 (8.3) | 0 (-1.2, 1.2) | 0.957 | -0.2 (-1.4, 0.9) | 0.679 | 974 |
|  | WASH | 245 | 19.9 (8.5) | 2 (0.5, 3.4) | 0.008 | 2.1 (0.7, 3.4) | 0.003 | 974 |
|  | WASH & IYCF | 246 | 18.1 (8.3) | 0.2 (-1.1, 1.4) | 0.812 | 0.4 (-0.6, 1.4) | 0.448 | 974 |

Table S5-2: Secondary blood pressure outcomes

### Table S6: Secondary growth outcomes

| Reactance at 50 kHz, Ohms | SoC | 243 | 72.7 (11.6) | No IYCF | 489 | 72.1 (10.7) | 0.0 (ref) |  | 0.0 (ref) |  |  |
| --- | --- | --- | --- | --- | --- | --- | --- | --- | --- | --- | --- |
|  | IYCF | 247 | 71.6 (10.9) | IYCF | 492 | 71.4 (10.3) | -0.8 (-2.3, 0.7) | 0.276 | 0.1 (-1.3, 1.6) | 0.858 | 971 |
|  | WASH | 246 | 71.6 (9.7) | No WASH | 490 | 72.1 (11.2) | 0.0 (ref) |  | 0.0 (ref) |  |  |
|  | WASH & IYCF | 245 | 71.1 (9.7) | WASH | 491 | 71.4 (9.7) | -0.6 (-2.1, 0.9) | 0.469 | -0.7 (-2.1, 0.6) | 0.263 | 971 |
| Resistance at 50 kHz, Ohms | SoC | 244 | 837.3 (97.8) | No IYCF | 491 | 833.2 (98.5) | 0.0 (ref) |  | 0.0 (ref) |  |  |
|  | IYCF | 250 | 828.2 (95.5) | IYCF | 495 | 826.4 (88.5) | -6.9 (-19, 5.1) | 0.26 | 2.1 (-8, 12.2) | 0.685 | 976 |
|  | WASH | 247 | 829.2 (99.1) | No WASH | 494 | 832.7 (96.7) | 0.0 (ref) |  | 0.0 (ref) |  |  |
|  | WASH & IYCF | 245 | 824.6 (80.8) | WASH | 492 | 826.9 (90.4) | -5.5 (-17.6, 6.5) | 0.368 | -5.5 (-16, 5) | 0.306 | 976 |

Table S6-1: Secondary growth outcomes

## Expanded methods

### Figure S1: Directed acyclic graph for adjusted models


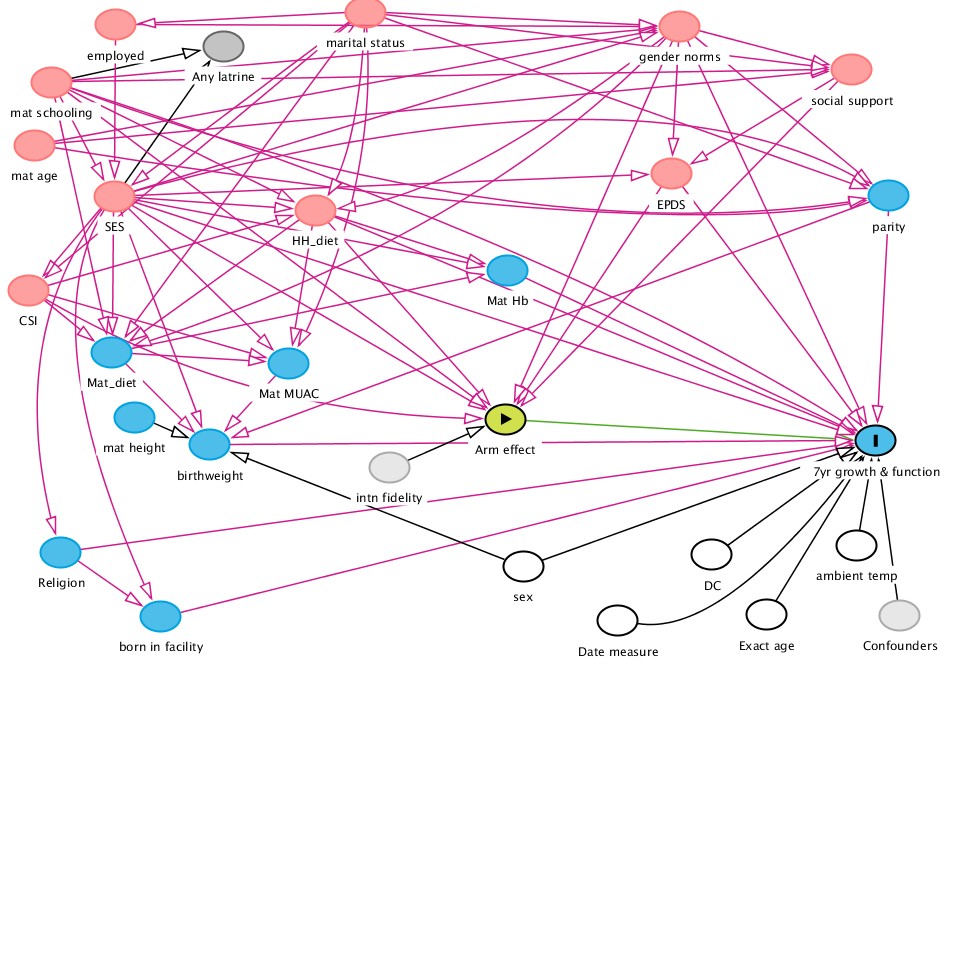


Figure S1: DAG exploring effect of intervention arm on 7 year growth and function for the SHINE study.

CSI: Coping strategies index, SES: wealth index score, mat age: maternal age, mat schooling: maternal schooling in years, HH_diet: household dietary diversity score, EPDS: Edinburgh Postnatal Depression Score ,employed: if mother employed, marital status, gender norms score, social support score, Mat Hb: Maternal haemoglobin, mat_diet: Maternal dietary diversity score, Mat MUAC: maternal MUAC score. Intervention fidelity and confounders were not measured.

Adjusted analysis models included calendar date measured defined by quarter, child age, ambient temperature, data collector, female sex, baseline depression score (EPDS), household dietary diversity score, maternal dietary diversity score, wealth index, birthweight, gender norms score, maternal education and parity.

### Validation and quality control of SAHARAN toolbox

The methods for the SAHARAN toolbox have been previously described^1^. For the SHINE Follow-up study, supportive supervision was provided by the project lead with monitored field visits every week, and additionally by the study clinician every 3 to 6 months (JDP). Every 6 to 9 months, data collectors conducted a standardisation exercise. There were eight Data collectors (DC’s), so standardisation was performed simultaneously on two children, each child in a separate homestead, using groups of four DC’s for each child.

Cognition

For cognition, the KABC-II was performed by one DC and three other DC’s simultaneously scored the same child independently whilst being blinded to each other’s marking. This was repeated for a different child each day over four days, so that each DC was observed once by the other DC’s and observed by the project lead and/or study clinician. A similar process for the SAT and timing for the finger tapping was also performed. The absolute value of intra-class correlation for average measures between data collectors for the same child was >0.99 for the scoring of the KABC-II and SAT and also for the timing of finger tapping.

Physical function

For physical function measurements, standardisation was not performed because the grip strength was recorded digitally. Similarly, the shuttle run test and broad jump distance could only be directly observed by the data collector performing the measurement, without independent measurement.

Anthropometry

Anthropometry standardisation sessions were also performed on the child in two groups of four DC’s each. However, this time anthropometry was measured twice by each DC, who remained blinded to the other DCs’ measurements. The anthropometry measurements were done firstly before the cognition measurements, and then the cognition measurements at the end of the visit. Anthropometric measurements conducted comprised height, head circumference, leg length, and triceps, calf, subscapular and suprailiac skinfold measurements. Weight and bioimpedance measurements were not included because these digital measurements were assumed to have minimal intra- and inter-operator error.

The intra-observer technical error of measurements (TEM) was calculated by finding the deviation between an operator’s individual measures on the same child between the morning and afternoon measurements in this calculation^2^

$$TEM=\sqrt{\frac{\Sigma(x_{a}-x_{p})_{i}^{2}}{2n}}$$

Where *x_a_ is the morning measurement and x_p_ is the afternoon measurement by the same DC on the same child. N = number of children measured, i= number of differences and* $\Sigma$ is the *sum of differences.*

The differences were then squared and then summed between all the children measured, dividing this by the number of children multiplied by 2, and then applying the square root. The inter-observer TEM was also calculated across all measurements as a comparison using this equation^3^.

$$TEM=\sqrt{\left( \left( \Sigma_{1}^{N}(\Sigma_{1}^{K}M^{2} \right)-\frac{\frac{\left( \Sigma_{1}^{K}M \right)^{2}}{K}}{N(K-1)} \right)}$$

Where N is the number of individuals measured (eg 8 children), K is the number of data collectors (eg 8 DC’s) and M is the measurement.

The relative TEM was also calculated to enable comparison of technical error between the measurement methods used^3^:

$$Relative TEM=\left( \frac{TEM}{Mean} \right) \times100$$

The measures selected included all of the different types of anthropometry equipment used. Comparing the TEM with the ChroSAM study^2^ standardisation showed a similar level of accuracy. Data collectors who showed higher TEM were supported with additional training and monitoring.

### Definition of disability

The Washington Group UNICEF tool^4^ was used for screening, and then confirmed with clinical notes from the team recorded on the caregiver questionnaire. For disability screening, a definition of functional difficulty was made for any answer that recorded ‘a lot of difficulty’ in sight, hearing, walking, self-care or communication. A definition of severe functional difficulty was made for any answer with ‘cannot do at all’ in sight, hearing, walking, self-care or communication. Similarly, a definition of learning difficulties or severe learning difficulties was defined for any answer to ‘a lot of difficulty’ or ‘cannot do at all’ for questions that asked if the child had difficulties in learning or remembering.

Definitions of disability were then confirmed by examining all additional comments recorded by the data collector. As part of training, data collectors were taught to record any additional disabilities with the child in the comments section of the case report form (CRF) for the child. Of note, the clinical notes section revealed two additional children who had a cognitive disability according to the written DC reports. For physical disability, the comments of the data collector determined whether the child was excluded from specific physical function tests due to specific problems (eg asthmatic so shuttle run test not performed). Of note, acute injuries that affected physical function were determined by DC written report as the WG UNICEF did not ask about acute injuries.

## List of abbreviations

| **Abbreviation** | **Explanation** |
| --- | --- |
| CHU | Children unexposed to HIV (or born to mothers without HIV) |
| CHW | Community Health workers who work within the catchment area of the SHINE households and are employed by the Zimbabwean Ministry of Health and Child Care. |
| CI | Confidence interval, usually set at 95%, which refers to a range of values where there is a 95% chance of the value being within this range. |
| CPRS | Child Parent Relationship Scale measure of nurturing |
| DAG | Directed Acyclic Graph |
| DC | Data Collector |
| EPDS | Edinburgh Postnatal Depression Score |
| GEE | Generalised estimating equations: the technique used to explore associations that can also account for clustering. |
| HAZ | Height-for-age Z-score |
| Hb | Haemoglobin, measured in grams/ decilitre (g/dl) |
| HIC | High income country as defined by world bank |
| IYCF | Infant and Young Child Feeding intervention: For SHINE this was a lipid based nutrient supplement for the child from age 6-18 months, together with monthly visits with nutrition advice. |
| Imp I | Impedance Index, measured by bioimpedance as a measure of relative lean mass, with units m^2^ Ohms^-1^ |
| IQR | Inter-quartile range: the range between the top and bottom quartile |
| KABC-II | The Kaufman Assessment Battery for children 2^nd^ edition. |
| LAZ | Length-for-age Z-score (typically measured <24 months) |
| LMI | Lean mass index, measured by bioimpedance as a measure of relative lean mass divided by height^2^, with units Ohms^-1^ |
| MLWH | Mothers living with HIV |
| MOHCC | The Zimbabwean Ministry of Health and Child Care, who works in partnership with Zvitambo |
| MRCZ | The Medical Research Council of Zimbabwe |
| MPI | Mental Processing index: the cognitive total for the Kaufman Assesment Battery for children, which was the primary outcome for this follow-up study. |
| MUAC | Mid-upper arm circumference, measured in cm. |
| Plus-EF | The Plus EF tablet-based executive function test combined 3 subtests of the multi-source interference subtest, stars and flowers subtest and flanker subtest. |
| SAHARAN | The School-Age Health, Activity, Resilience, Anthropometry and Neurocognitive (SAHARAN) toolbox includes all the school-age outcomes measured |
| SAP | The pre-specified statistical analysis plan, available on Open Science Framework at <https://osf.io/8e2zh> |
| SAT | School Achievement Test: the total includes the sum of the 3subtests that measure numeracy, reading and writing. |
| SD | Standard deviation: a measure of the spread of the data |
| SDQ | Strengths and Difficulties Questionnaire: A 25-item caregiver reported questionnaire which details the child’s socioemotional function. 20 of the questions form the problem-based total SDQ total reported in the paper. Each subscale is detailed in supporting information |
| SFU | SHINE Follow-up: the abbreviation for the SHINE children followed up at 7 years. |
| SHINE | Sanitation Hygiene Infant Nutrition Efficacy cluster randomised trial |
| SOC | Standard of Care arm in the SHINE Trial. All trial arms including SOC had breastfeeding support to 6 months and additional support for PMTCT screening. |
| SQ-LNS | Small quantity lipid based nutrient supplementation are designed to complement the diets of children aged 6 months and older by including multiple micronutrients within a food base that also provides energy, protein and essential fatty acids. |
| WASH | Water, Sanitation and Hygiene intervention: For SHINE this included a latrine, field washing stations, Chlorine water treatment and monthly visits with hygiene advice. |
| WASH + IYCF | The combined WASH and IYCF intervention within the SHINE trial |
| WAZ | Weight-for-age Z-score |
| WG | Washington Group UNICEF tool for screening for child disability |
| Zvitambo | The Zvitambo Institute for Maternal and Child Health Research previously coordinated the SHINE trial and now works on a variety of projects in partnership with the MOHCC. |

## References

1. Piper JD, Mazhanga C, Mapako G, et al. Characterising school-age health and function in rural Zimbabwe using the SAHARAN toolbox. *PLOS ONE* 2023; **18**(5): e0285570.

2. Lelijveld N. Long-term effects of severe acute malnutrition on growth, body composition, and function; a prospective cohort study in Malawi; 2016.

3. Moss C, Kuche D, Bekele TH, et al. Precision of Measurements Performed by a Cadre of Anthropometrists Trained for a Large Household Nutrition Survey in Ethiopia. *Curr Dev Nutr* 2020; **4**(9): nzaa139.

4. Cappa C, Mont D, Loeb M, et al. The development and testing of a module on child functioning for identifying children with disabilities on surveys. III: Field testing. *Disabil Health J* 2018; **11**(4): 510-8.
