# Supplementary material for: School-age growth and development following infant feeding and/or water, sanitation, and hygiene interventions in rural Zimbabwe: long-term follow-up of a cluster-randomised trial
Source: eClinicalMedicine. 2024 Nov 22;78:102946. doi: 10.1016/j.eclinm.2024.102946 (PMC11617972; doi:10.1016/j.eclinm.2024.102946)
Supplement: CONSORT_ROUTINE_checklist [file mmc2.docx]

Table 2

Combined CONSORT 2010 and CONSORT-ROUTINE checklist

| **Section/topic** | **Item No** | **CONSORT extension for trials conducted using cohorts or routinely collected data item** | **Reported on page No** |
| --- | --- | --- | --- |
| **Title and abstract** | | | |
|  | 1a | Identification as a randomised trial in the title | 1 |
|  | 1b | Structured summary of trial design, methods, results, and conclusions (for specific guidance see CONSORT for abstracts). Specify that a cohort or routinely collected data were used to conduct the trial and, if applicable, provide the name of the cohort or routinely collected database(s) | 2 |
| **Introduction** | | | |
| Background and objectives | 2a | Scientific background and explanation of rationale | 7 |
|  | 2b | Specific objectives or hypotheses | 8 |
| **Methods** | |  |  |
| Trial design | 3a | Description of trial design (such as parallel, factorial) including allocation ratio, that a cohort or routinely collected database(s) was used to conduct the trial (such as electronic health record, registry) and how the data were used within the trial (such as identification of eligible trial participants, trial outcomes) | 9 |
|  | 3b | Important changes to methods after trial commencement (such as eligibility criteria), with reasons |  |
| Cohort or routinely collected database | ROUTINE-1 | Name, if applicable, and description of the cohort or routinely collected database(s) used to conduct the trial, including information on the setting (such as primary care), locations, and dates (such as periods of recruitment, follow-up, and data collection) | 9 |
|  | ROUTINE-2 | Eligibility criteria for participants in the cohort or routinely collected database(s) | 10 |
|  | ROUTINE-3 | State whether the study included person-level, institutional-level, or other data linkage across two or more databases and, if so, linkage techniques and methods used to evaluate completeness and accuracy of linkage |  |
| Trial participants | 4a | Eligibility criteria for trial participants, including information on how to access the list of codes and algorithms used to identify eligible participants, information on accuracy and completeness of data used to ascertain eligibility, and methods used to validate accuracy and completeness (eg, monitoring, adjudication), if applicable | 9, 11 |
|  | 4b | Settings and locations where the data were collected | 11 |
|  | ROUTINE-4 | Describe whether and how consent was obtained |  |
| Interventions | 5 | The interventions for each group with sufficient details to allow replication, including how and when they were actually administered | 9, 10 |
| Outcomes | 6a | Completely defined pre-specified primary and secondary outcome measures, including how and when they were ascertained and the cohort or routinely collected database(s) used to ascertain each outcome | 10, 12 |
|  | ROUTINE-5 | Information on how to access the list of codes and algorithms used to define or derive the outcomes from the cohort or routinely collected database(s) used to conduct the trial, information on accuracy and completeness of outcome variables, and methods used to validate accuracy and completeness (eg, monitoring, adjudication), if applicable | 13, |
|  | 6b | Any changes to trial outcomes after the trial commenced, with reasons |  |
| Sample size | 7a | How sample size was determined | 14 |
|  | 7b | When applicable, explanation of any interim analyses and stopping guidelines |  |
| Sequence generation | 8a | Method used to generate the random allocation sequence |  |
|  | 8b | Type of randomisation; details of any restriction (such as blocking and block size) | 9, 11 |
| Allocation concealment mechanism | 9 | Mechanism used to implement the random allocation sequence (such as embedding an automated randomiser within the cohort or routinely collected database(s)), describing any steps taken to conceal the sequence until interventions were assigned | 11 |
| Implementation | 10 | Who generated the random allocation sequence, who enrolled participants, and who assigned participants to interventions | 11 |
| Blinding | 11a | If done, who was blinded after assignment to interventions (for example, participants, care providers, those assessing outcomes) and how | 2, 13 |
|  | 11b | If relevant, description of the similarity of interventions | 17, 25 |
| Statistical methods | 12a | Statistical methods used to compare groups for primary and secondary outcomes | 11, 13,14 |
|  | 12b | Methods for additional analyses, such as subgroup analyses and adjusted analyses | 3, 14 |
| **Results** | | | |
| Participant flow (a diagram is strongly recommended) | 13a | For each group, the number of participants in the cohort or routinely collected database(s) used to conduct the trial and the numbers screened for eligibility, randomly assigned, offered and accepted interventions (eg, cohort multiple RCTs), received intended treatment, and analysed for the primary outcome | 16, 23 |
|  | 13b | For each group, losses and exclusions after randomisation, together with reasons | 23 |
| Recruitment | 14a | Dates defining the periods of recruitment and follow-up | 9, 16, |
|  | 14b | Why the trial ended or was stopped | 9 |
| Baseline data | 15 | A table showing baseline demographic and clinical characteristics for each group | 25, 26 |
| Numbers analysed | 16 | For each group, number of participants (denominator) included in each analysis and whether the analysis was by original assigned groups | 16, 4 |
| Outcomes and estimation | 17a | For each primary and secondary outcome, results for each group, and the estimated effect size and its precision (such as 95% confidence interval) | 27 to 34 |
|  | 17b | For binary outcomes, presentation of both absolute and relative effect sizes is recommended |  |
| Ancillary analyses | 18 | Results of any other analyses performed, including subgroup analyses and adjusted analyses, distinguishing pre-specified from exploratory | 18, Appendix |
| Harms | 19 | All important harms or unintended effects in each group (for specific guidance see CONSORT for harms) | 16 |
| **Discussion** | | | |
| Limitations | 20 | Trial limitations, addressing sources of potential bias, imprecision, and, if relevant, multiplicity of analyses | 21 to 22 |
| Generalisability | 21 | Generalisability (external validity, applicability) of the trial findings | 20 |
| Interpretation | 22 | Interpretation consistent with results, balancing benefits and harms, and considering other relevant evidence, including the implications of using data that were not collected to answer the trial research questions | 19 to 22 |
| **Other information** | | | |
| Registration | 23 | Registration number and name of trial registry | 2 |
| Protocol | 24 | Where the full trial protocol can be accessed, if available | 11 |
| Funding | 25 | Sources of funding and other support for both the trial and the cohort or routinely collected database(s), role of funders | 3 |

RCT=randomised controlled trial.

This checklist is also presented separately in web table 1.
